# A novel polypeptide CAPG-171aa encoded by circCAPG plays a critical role in triple-negative breast cancer

**DOI:** 10.1186/s12943-023-01806-x

**Published:** 2023-07-05

**Authors:** Runjie Song, Peilan Guo, Xin Ren, Lijun Zhou, Peng Li, Nafis A Rahman, Sławomir Wołczyński, Xiru Li, Yanjun Zhang, Mei Liu, Jiali Liu, Xiangdong Li

**Affiliations:** 1 State Key Laboratory of Agrobiotechnology, College of Biological Sciences, China Agricultural University, Beijing, 100193 China; 2 Department of Physiology, Institute of Biomedicine, University of Turku, Turku, Finland; 3 Department of Reproduction and Gynecological Endocrinology, Medical University of Bialystok, Bialystok, Poland; 4 Department of General Surgery, Chinese PLA General Hospital, Beijing, 100071 China; 5 Department of Pathology, Chinese PLA General Hospital, Beijing, 100071 China

**Keywords:** TNBC, CircCAPG, CAPG-171aa, STK38, MEKK2, *SLU7*, Therapeutic target

## Abstract

**Background:**

The treatment of Triple-negative breast cancer (TNBC) has always been challenging due to its heterogeneity and the absence of well-defined molecular targets. The present study aims to elucidate the role of protein-coding circRNAs in the etiology and carcinogenesis of TNBC.

**Methods:**

CircRNA expression data in TNBC (GEO: GSE113230, GSE101123) were reanalyzed and then circCAPG was selected for further study. To identify the polypeptide-coding function of circCAPG, a series of experiments, such as Mass spectrometry and dual-luciferase reporter assays were conducted. Cell proliferation, apoptosis and metastasis parameters were determined to investigate the cancerous functions CAPG-171aa plays in both TNBC organoids and nude mice. Mechanistically, the relation between CAPG-171aa and STK38 in TNBC was verified by immunoprecipitation analyses and mass spectrometry. The interactions between *SLU7* and its binding site on circCAPG were validated by RIP-qPCR experiments.

**Results:**

In both TNBC clinical samples and cell lines, the expression level of circCAPG was identified to be higher compared with normal ones and positively correlated with the overall survival (n = 132) in a 10-year follow-up study, in which the area under the curve of receiver operating characteristic was 0.8723 with 100% specificity and 80% sensitivity. In addition, we found that circCAPG knockdown (KD) significantly inhibited the growth of TNBC organoids. Intriguingly, circCAPG can be translated into a polypeptide named CAPG-171aa which promotes tumor growh by disrupting the binding of serine/threonine kinase 38 (STK38) to SMAD-specific E3 ubiquitin protein ligase 1 (SMURF1) and thereby preventing MEKK2 ubiquitination and proteasomal degradation. Furthermore, we found that *SLU7* Homolog- Splicing Factor (*SLU7*) can regulate the bio-generation of circCAPG through binding to the flanking Alu sequences of circRNA transcripts.

**Conclusions:**

circCAPG significantly enhances the proliferation and metastasis of TNBC cells by encoding a novel polypeptide CAPG-171aa and afterwards activates MEKK2-MEK1/2-ERK1/2 pathway. Additionally, the formation of circCAPG is found to be mediated by *SLU7*. The present study provides innovative insight into the role of protein-coding circRNAs CAPG-171aa in TNBC, and its capacity to serve as a promising prognostic biomarker and potential therapeutic target in TNBC.

**Graphical abstract:**

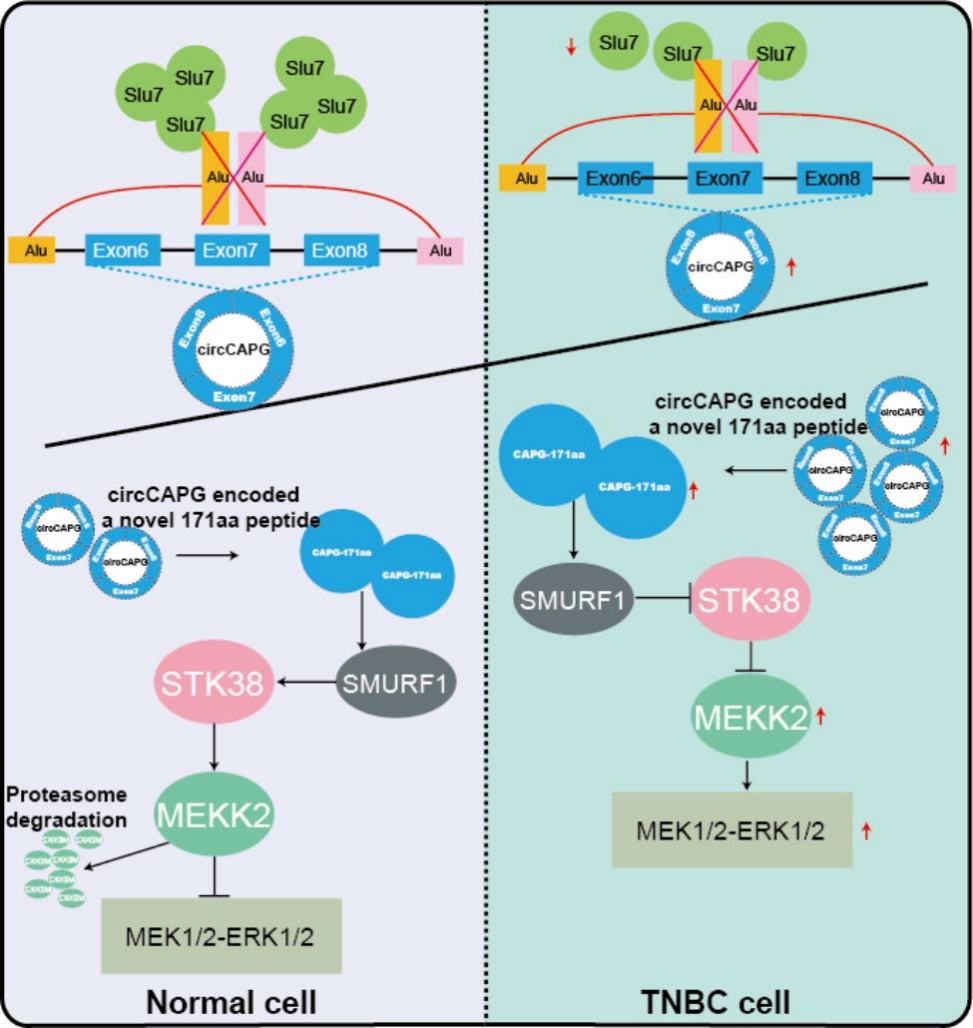

**Supplementary Information:**

The online version contains supplementary material available at 10.1186/s12943-023-01806-x.

## Highlights

1) circCAPG is significantly highly expressed in TNBC.

2) The knockdown (KD) of circCAPG significantly inhibits the growth of TNBC patient-derived organoids.

3) circCAPG can be translated into a polypeptide named CAPG-171aa.

4) CAPG-171aa prevents MEKK2 ubiquitination and proteasomal degradation.

5) *SLU7* regulates the bio-generation of circCAPG.

In this model, *SLU7*-mediated circCAPG is identified to encode a polypeptide CAPG-171aa which promotes tumor growth by disrupting the binding of serine/threonine kinase 38 (STK38) to SMAD-specific E3 ubiquitin protein ligase 1 (SMURF1) and thereby preventing MEKK2 ubiquitination and proteasomal degradation.

**Supplementary Information:**

The online version contains supplementary material available at 10.1186/s12943-023-01806-x.

## Background

Breast cancer (BC) has become “the world’s largest cancer” shown in “2020 Global Cancer Report” and its incidence rate is growing rapidly recent year [1]. Notably, triple-negative breast cancer (TNBC) accounting for 15–20% of all BC subtypes is the most malignant subtype with severe proliferative and aggressive phenotype [2]. TNBC is characterized by deficient expression of estrogen receptor (ER), progesterone receptor (PR) and human epidermal growth factor receptor type 2 (Her-2) [3]. Therefore, unlike the hormone receptor-positive and Her-2-positive subtypes, TNBC still lacks valid clinical biomarkers and therapeutic strategies [4]. The effort to screen out reliable drug targets to improve the prognosis of TNBC patients is urgent.

circRNAs were covalently closed noncoding RNAs without 5’cap or polyadenylated tail. circRNAs are resistant to exonucleolytic degradation due to their closed-loop structure. Thus, circRNAs are more stable than their linear counterparts. Amount of studies have demonstrated that circRNAs can be detected in plasma, saliva, and exosomes [5–8]. For example, Ju et al., [9] developed a prognostic tool based on four circRNAs to improve the prognostic stratification of patients with curatively resected colon cancer at stage II/III. All these provide strong support for the practice of circRNAs as biomarkers or therapeutic targets in clinical treatment.

Majority of studies indicate that circRNAs mainly function as the “sponges” of microRNA/protein in various cancers [7, 10, 11], including TNBC [5, 12, 13]. Apart from these, both our group [7, 14] and other teams [6, 15] have demonstrated the reality of circRNA-encoded proteins, which suggests their potential oncogenic molecular actions in disease development. The study of the protein-coding ability of circRNAs provides novel insight for clarifying the etiology of tumors and is becoming a hot topic. For example, EIF6-224aa, encoded by circ-EIF6, promotes TNBC progression via stabilizing MYH9 and activating Wnt/beta-catenin pathway [6]. HER2-102, encoded by circ-HER2, promotes TNBC progression via activating EGFR/HER3 signaling [15]. However, whether other protein-coding circRNAs involve in TNBC and how they work in TNBC remain elusive. Considering the unique translatable functions of circRNAs in oncogenic pathways, a better understanding of the molecular action of circRNAs in TNBC could help us figure out creative drug targets for the clinical treatment. After mining the public database, we screened out a circRNA called circCAPG (circBase ID: hsa_circ_0055412), which is highly expressed in TNBC and positively correlated with both advanced clinical stage and poor prognosis. More importantly, we found that circCAPG encoded a 171-aa peptide (CAPG-171aa) which plays crucial roles in tumor growth and metastasis. Therefore, in this study, we aimed to elucidate the molecular action of CAPG-171aa, the mechanism of circCAPG biogenesis, and explore the potential therapeutic capacity of this novel polypeptide CAPG-171aa in the TNBC.

## Materials and methods

### Patients and tissue samples

The paraffin-embedded (FFPE) TNBC (n = 132) and adjacent tissues of TNBC (n = 40) used in this study were provided by TNBC patients undergoing surgical resection at the Chinese PLA General Hospital. Clinical data was obtained from medical records and follow-up information was derived from telephone consultations or death certificates. The whole study was authorized by the Ethics Committee of the Chinese PLA General Hospital and all research complied with the principles of the Declaration of Helsinki. The study was approved by the Ethics Committee of Chinese PLA General Hospital and all the patients had signed the informed consent.

### Bioinformatic analysis

circRNA expression data in TNBC (GEO: GSE113230, GSE101123) was obtained from the NCBI GEO database and annotated in circBase. TNBC mRNA data obtained from the RTCGA package, EdgeR, and DEseq2 package (version 3.12.1) was used to detect differentially expressed circRNAs or mRNAs. Multiple changes > 1.3 and P values < 0.05 were considered as significant differences. The ggboxplot, ggplot2 and pheatmap packages in R were used to display boxplot, volcano, and heatmaps.

### Cell culture

All cell lines were purchased from Procell Life Science & Technology Co., Ltd (Wuhan, China) and characterized by DNA fingerprinting and passaged < 6 months. MDA-MB-231 and MDA-MB-468 cells were grown in Leibovitz’s L-15 Medium (KeyGEN, KGM41300N-500) supplemented with 10% fetal bovine serum (FBS, Gibco, USA). HEK293T, Hs578T, MCF7, and T47D cells were cultured with Dulbecco’s modified Eagle’s medium (DMEM, Gibco, USA) supplemented with 10% FBS. ZR-75-1 cells were cultured with Minimum Essential Medium (KeyGEN, KGM41500N-500) supplemented with 10% FBS. MCF-10 A cells were grown in DMEM/F12 medium (Gibco, USA) supplemented with 5% horse serum (Solarbio, China) and growth supplements. All cell lines were cultured in 5% CO_2_ at 37 °C in a humidified atmosphere.

### Human TNBC organoid model

Fresh specimens of TNBC tissues were cut into ~ 1mm^3^ fragments on ice and digested for 40 min at 37 ℃ with 220 rpm. The digestion medium was added DMEM/F12 supplemented with Y-27,632 (10 µM), primocin, and collagenase II (1 mg/mL). The tissues were terminated by FBS after digestion. The suspension liquid was filtered with a 100 μm filter strainer and centrifuged at 450 g for 5 min at 8℃. Red blood cells in the cell pellet are lysed using red blood cell lysate and centrifuged at 450 g for 5 min at 8 ℃. Subsequently, wash the pellet three times using D-BSA. Resuspended with organoid medium and homogeneously seeded in microwell molds.

### Reverse transcriptase PCR (RT-PCR) and quantitative real-time PCR (qRT-PCR)

Total RNA was extracted from TNBC cell lines and tissue specimens with Trizol (Yeasen, 19211ES60) and a total of 1.5 µg/sample of RNA was used for cDNA synthesis with M-MLV RT Kit with gDNA Clean for qPCR (Accurate Biotechnology(Hunan)Co., Ltd, AG11705) and TransScript® Fly First-Strand cDNA Synthesis SuperMix for RT-PCR (TransGen Biotech, AF301) following the procedure of 42 °C for 90 min and 95 °C for 5 min. RT-PCR and qRT-PCR were performed with 2×Taq PCR StarMix (GENSTAR, A012) and 2X SYBR Green qPCR Master Mix (APExBIO, K1070), respectively. The relative expression levels of target genes were calculated following the formula of 2^−ΔΔCt^. All primers were synthesized by Tsingke Biological Technology (Tsingke, China). The primer sequences were listed in Table S1.

### Small interfering RNA (siRNA) and plasmid transfection

The siRNA specifically targeting STK38, EIF4A3, RBM38, HNRNPL, and circCAPG, as well as non-specific si-control were synthesized at RiboBio (Guangzhou, China). Two specific shRNAs, sh-circCAPG and sh-scramble, were cloned into PLKO.1-TRC plasmid (Tsingke, China), respectively, to silence the circCAPG, and sh-scramble works as a negative control.

To construct the circCAPG expression vector, the sequence of circCAPG was amplified from HEK293T cDNA using KeyPo Master Mix (Vazyme, PK511) and cloned into circular RNA expression plasmid PLO5-ciR (Geneseed, China). The circCAPG-flag plasmid was derived by inserting the flag sequence immediately to the upstream stop codon of the putative open reading frame (ORF) of circCAPG. The mutant of the internal ribosome entry site (IRES) of circCAPG (the IRES was deleted) was cloned into PLO5-ciR plasmid to generate circCAPG-flag-IRES-mut. To construct a circCAPG vector containing flanking intron sequence, the genomic region of circCAPG with its flanking introns was synthesized by Tsingke Biological Technology (Tsingke, China) and inserted into the pCDNA3.1 vector. Full length of UBA52, PRPF31, ATP5A1, TUFM, STK38, GTF21, PRPFKB3, UQCRC2, PPM1B, KHDC1, SNRPA1, NPM1, NCBP2, LSM5 and SLU7 was amplified from HEK293T cDNA using 2×TransStart® FastPfu PCR SuperMix (TransGen Biotech, AS221) and cloned into pCDNA3.1 vector. To assay IRES activity of circCAPG, the promoter region of F-Luc in the psiCHECK2 vector was deleted and the IRES sequences of circCAPG were cloned behind R-Luc. All plasmids were extracted using an endotoxin-free plasmid extraction kit (Shandong Sparkjade Biotechnology Co., Ltd., AD0105). Primer sequences were listed in Table S1. All expression vector has been verified by sequencing (Sangon Biotech, China). All transfection experiments were conducted with Lipofectamine™ 3000 Transfection Reagent (Invitrogen, USA) following the manufacturer’s instructions.

### Actinomycin D (ACTD) assay

For the ACTD assay, the extracted total RNAs were treated with 1 µg/mL actinomycin D (Sigma-Aldrich, USA) against new RNA synthesis for 0, 2, 4, 8, 12 and 24 h, respectively.

### Nucleocytoplasmic separation

The RNA of nuclear and cytoplasmic fractions of TNBC cells were extracted using a Paris kit (Invitrogen, USA) according to the manufacturer’s instructions.

### Virus production, infection, and puromycin selection

Vectors (sh-circCAPG-1, sh-circCAPG-2, sh-scramble, PLO5-ciR, circCAPG, circCAPG-flag, circCAPG-flag-IRES-mut, circCAPG-flag-ATG-mut) were transfected into 293T cells with Lipofectamine™ 3000 based on the manufacturer’s instructions. Infectious supernatant was collected twice after 48 and 72 h and then filtered through 0.45 μm filters (Hangzhou Cobetter Filtration Equipment Co., Ltd). MDA-MB-231 and MDA-MB-468 cells were infected with the appropriate amount of the virus recombinant lentivirus for 48 h and then selected by 1 and 2 µg/mL puromycin (Sigma-Aldrich, USA), respectively, for 72 h.

### Cell proliferation, colony formation, and wound-healing assay

MDA-MB-231 and MDA-MB-468 cells (1 × 10^3^) were seeded into 96-well plates and cell viability was determined by absorbance at 450 nm after 0, 24, 48, 72 and 96 h with Cell Counting Kit-8 (Elabscience, E-CK-A362). For colony formation assay, cells were seeded in 6-well plates (SORFA Life Science) at a density of 3 × 10^3^ cells per well and incubated in L15 containing 10% FBS for two weeks. Then, the cells were washed with ice-cold PBS twice, fixed with formaldehyde, and stained with a crystal violet staining solution (Beyotime, China). For the wound-healing assay, cells were seeded in a 24-well plate (SORFA Life Science) with a serum-free medium. Then, a sterile 10 µL plastic pipette tip (CellProBio) was used to scratch through the single-cell layer, and images were captured after 0 and 48 h at the same place with a microscope (Nikon, Japan).

### Migration and invasion assays

Migration and invasion assays were performed using the Transwell system (Nest, 723,001). For the 48 h migration assay, MDA-MB-231 and MDA-MB-468 cells (1 × 10^5^) were seeded in small chambers (Nest, 723,001) with a serum-free medium, and a 700 mL medium with 20% FBS was added to the bottom wells. Likewise, for invasion assay, 1 × 10^5^ cells were seeded in Matrigel-coated chambers with serum-free medium and cultured for 48 h along with L15 supplemented with 20% FBS in the bottom wells. The images were captured by microscope (Nikon, Japan).

### Immunofluorescence assay

For immunofluorescence assay, TNBC cells were collected and fixed with ice cold acetone. The cells were fixed with 4% paraformaldehyde for 30 min at room temperature and then treated with 0.2% Trition X-100 for 10 min. Cells were incubated with 10% goat serum for 60min at 37°C and incubated with the KI67 antibodies at 4°C overnight. After washing with PBS, cells were incubated with corresponding fluorescent secondary antibody and counterstained for nuclei using 4’, 6-diamidino-2-phenylindole (DAPI) (Solarbio, China) for 15 min at room temperature.

### Luciferase reporter assay

For IRES activity analysis, 293T cells were transfected with psiCHECK2 containing circCAPG IRES. After 72 h transfection, the luciferase activity of circCAPG IRES was assessed using the dual-luciferase reporter kit (TransGene, China) based on the manufacturer’s instructions.

### RIP assays

RIP assay was carried out with the PureBinding®RNA Immunoprecipitation Kit (GENESEED, P0101), and the anti-SLU7 antibody (Proteintech, USA) and IgG antibody (Millipore, USA) were utilized according to the manufacturer’s instructions. Briefly, protein A/G magnetic beads were incubated with anti-SLU7 or IgG, and negative control antibodies, respectively, and then incubated with cells. Subsequently, RNA was co-precipitated and extracted, and finally quantitated by RT-qPCR.

### Ubiquitination assay

MDA-MB-231 and MDA-MB-468 cells were treated with 10 µg/mL MG132, respectively, (Solarbio, IM0310) for 12 h. Cell lysates were obtained using PierceTM IP lysis buffer (Thermo Fisher Scientific, USA) supplemented with a cocktail (Thermo Fisher Scientific, USA) and then incubated with anti-MEKK2 antibody (Proteintech, USA) and Protein A/G beads overnight at 4℃. Finally, protein A/G beads with the bound immunoprecipitates were collected and analyzed using Western blotting.

### Cycloheximide (CHX) chase assay

The MDA-MB-231 and MDA-MB-468 cells in each group were treated with 10 µg/mL CHX (Sigma-Aldrich, USA) for 0, 1, 2, 4, and 8 h. The expression level of MEKK2 protein was determined by Western blotting.

### Co-immunoprecipitation

Immunoprecipitation and co-immunoprecipitation were carried out with Pierce Classic Magnetic IP/ Co-IP Kit (TermoFisher Scientific, USA) according to the manufacturer’s instructions. Cells were washed with ice-cold PBS and lysed in cold PierceTM IP lysis buffer. Then, the supernatant was collected and incubated with a specific IP antibody or negative control IgG at 4 °C overnight. Next, the co-immunoprecipitates were separated and analyzed by SDS–PAGE, MS, or Western blotting. VeriBlot (Abcam, USA) was used to avoid the detection of heavy and light chains. Finally, protein A/G beads with the bound immunoprecipitates were collected and analyzed using Western blotting.

### Protein isolation and western blotting

Cells were lysed with protein lysis buffer (Solarbio, R0020) containing cocktail and then separated by SDS-PAGE gels, and transferred onto the polyvinylidene fluoride (PVDF) membrane (Millipore, USA). After being blocked with 5% nonfat milk, the membrane was incubated with a specific primary antibody at room temperature for 1 h, and then washed and incubated with a secondary antibody (Proteintech, USA) for 1 h. Western blotting was performed by anti-MEKK2 (Proteintech, 55106-1-AP), MEK1/2 (ABclonal, A4868), SMURF1 (Bioss, bs-9391R), STK38 (Huabio, ER60144), ERK1/2 (Proteintech, 11257-1-AP), UB (Cell Signaling Technology, #3936), Phospho-MEK1/2 (Ser221) (Cell Signaling Technology, #2338), Phospho-p44/42 MAPK (Erk1/2) (Thr202/Tyr204) (Cell Signaling Technology, #8544), CAPG (Bioss, bsm-60,451 M), CDK4 (AtaGenix Laboratories Co., Ltd.(Wuhan), Wuhan, PR China, PA9437H), Cyclin D1 (Yeasen, 30026ES50), BCL2 (Shanghai hengyuan biological technology co., LTD, A-01203), CAPG (Proteintech, 10194-1-AP), FLAG (SMART, /smart-lifesciences, SLAB0101), HA (Yeasen, 30702ES20), HIS (Yeasen, 30401ES10). β-ACTIN, GAPDH, or TUBULIN (Proteintech, USA) were used as loading controls. All antibodies were diluted as required by the manufacturer (New Cell & Molecular Biotech, WB100D). The antibodies used in this study are listed in Table S2.

### In vivo xenograft assay

The 4–5 weeks-old female BALB/c nude (nu/nu) mice purchased from SPF (Beijing) Biotechnology Co., Ltd. (Beijing, China) were housed under specific pathogen-free (SFP) conditions. Approximately 1 × 10^7^ / mL MDA-MB-231 cells in PBS supplemented with Matrigel (Yeasen, 40183ES10) (1:1) were injected subcutaneously into the BALB/c nude (nu/nu) mice after one week. All animal studies were approved by the Ethics Committee for Animal Experimentation of China Agricultural University.

### Statistical analysis

Two-tailed Student’s t-test was performed to calculate statistical significance with GraphPad Prism 8 (version 8.0.1, USA). Asterisks denote statistical significance (*P < 0.05, **P < 0.01, ***P < 0.001) and ns indicate no significance among groups. All data were validated in at least three independent experiments and represented as mean ± standard error of the mean (SEM).

## Results

### Identification and characteristics of circCAPG in TNBC

**Fig. 1:**
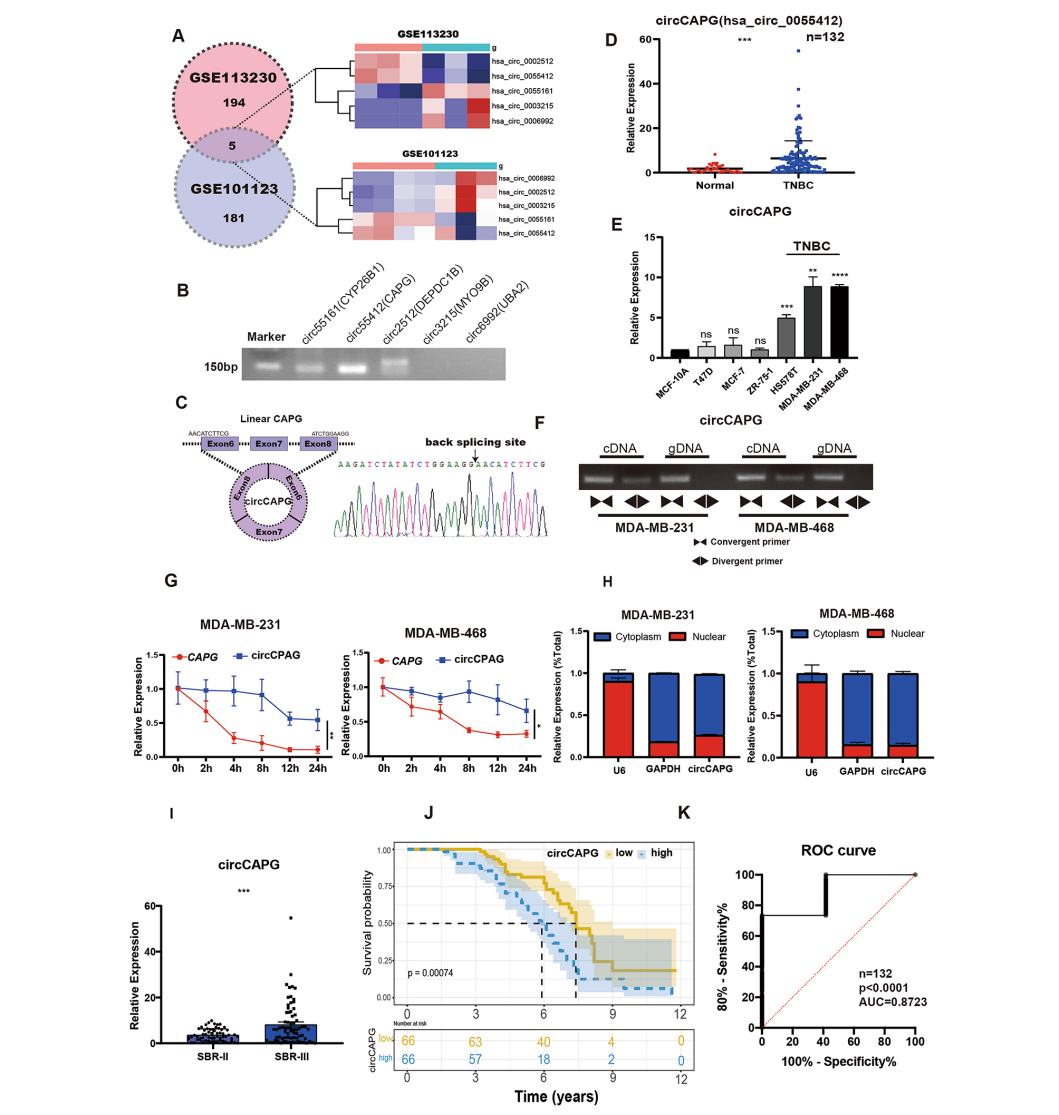
CircCAPG is highly expressed in TNBC. **(A)** Differentially expressed circRNAs in TNBC were analyzed based on the GEO database. Five circRNAs were identified to simultaneously exist in both GSE113230 and GSE101123 and thus selected. **(B)** RT-PCR analysis of five circRNAs in TNBC cell lines. **(C)** Schematic illustration of circCAPG conformation. The exon 6, exon 7, and exon 8 of CAPG mRNA formed circCAPG through back splicing. RNA sequencing of the back-splicing site of circCAPG was shown below. **(D)** qPCR of circCAPG in TNBC and adjacent tissues of TNBC. n = 132. **(E)** qPCR of circCAPG in various TNBC cell lines. **(F)** PCR products of circCAPG in cDNA and gDNA amplified using convergent or divergent primers in MDA-MB-231 and MDA-MB-468. **(G)** The half-life of linear CAPG and circCAPG in MDA-MB-231 and MDA-MB-468. **(H)** The distribution of circCAPG in the nuclear and cytoplasmic fraction. U6 and GAPDH were used as nuclear and cytoplasmic markers, respectively. **(I)** qPCR of circCAPG in TNBC tissue at different stages. **(J)** Survival analysis of 132 TNBC patients. The cutoff of ‘low’ and ‘high’ expression levels of circCAPG was decided according to the median of the circCAPG expression in TNBC tissue. **(K)** ROC curve of the diagnostic value of circCAPG. All data were representative of at least three biological replicates and shown as mean ± SEM. *P < 0.05, **P < 0.01, ***P < 0.001

To identify and characterize circular RNAs in TNBC, we analyzed microarray and RNA-seq datasets from the GEO database (GSE113230 and GSE101123) betweent TNBC and the adjacent noncancerous tissue. By using bioinformatics analysis, we obtained five circRNA candidates significantly differentially expressed in TNBC (Fig. 1A; Fig. S1A). To verify these 5 circRNAs, we carried out reverse transcription-PCR (RT-PCR) analyses with divergent primers in MDA-MB-231 cell line (Fig. 1B). Sanger sequencing confirmed three out of five circRNA candidates produced in ‘back-splicing’ manner (Fig. 1B and C). Of note, the expression level of circCAPG was the most significantly upregulated among these five circRNAs in TNBC (Fig. 1D; Fig. S1B). In addition, we found that among different subtypes of breast cancer, circCAPG was significantly highly expressed only in TNBC cell lines (Fig. 1E). Based on these data, our study focused on circCAPG.

Inspection of circCAPG’s maternal genomic region revealed that circCAPG was formed from exon 6–8 of Macrophage-capping protein (CAPG) gene (Fig. S1C). The complementary DNA (cDNA) and genomic DNA (gDNA) templates of TNBC cell lines were used as templates for RT-PCR. Results showed that circCAPG could only be amplified with cDNA template when using divergent primers but successfully amplified with either cDNA or gDNA templates when using convergent primers (Fig. 1F), which indicates the specific back-splicing manner of circCAPG. Actinomycin D assay validated the stability of circCAPG. Consistently, quantitative reverse transcription PCR (RT-qPCR) results revealed longer half-life of circCAPG compared with CAPG mRNA (Fig. 1G). Furthermore, the practice of random or oligo dT primers further confirmed the circular characteristics of circCAPG. Compared with random primers, the relative expression level of circCAPG was markedly decreased when using the oligo dT primers, whereas the expression of CAPG mRNA was not altered (Fig. S1D) indicating that circCAPG had no poly (A) tail. Additionally, nucleocytoplasmic separation assay revealed the cytoplasmic localization of circCAPG in TNBC cells (Fig. 1H).

To further evaluate the association between circCAPG expression and the clinical pathological features, RT-qPCR was performed in tumor (n = 132) and adjacent tissues of TNBC patients. We found that circCAPG was higher expressed in more malignant TNBC tissue that at advanced stage (Fig. 1I). Clinical data revealed that the expression level of circCAPG is significantly correlated with the tumor size, lymphatic invasion and TNM stage of TNBC (Table 1). Additionally, higher expression level of circCAPG accompanied with worse prognosis in TNBC patients shown in Kaplan-Meier survival analysis (Fig. 1J). Notably, the clinical value of circCAPG analyzed by receiver operating characteristic (ROC) curve showed that the specificity and sensitivity of diagnosis were 100% and 80%, respectively (Fig. 1K).

**Table 1:** Association between circCAPG expression and clinical featuresof TNBC

		circCAPG expression		
Variable	Cases (n = 132)	Low	High	p Value
**Age (Years)**				
≥ 50	72 60	41	31	0.3265
< 50		26	34	
**Tumor Size (cm)**				
≥ 2	63	35	28	0.0357*
< 2	69	32	37	
**Histologic grade**				
I + II	61	29	32	0.0003***
III	71	38	33	
**N status**				
Negative	76	35	41	0.0166*
Positive	56	32	24	
**Ki67 expression**				
Negative	76	35	41	0.0058**
Positive	56	32	24	

### The circCAPG knockdown (KD) inhibited cell proliferation, migration and invasion of TNBC

**Fig. 2:**
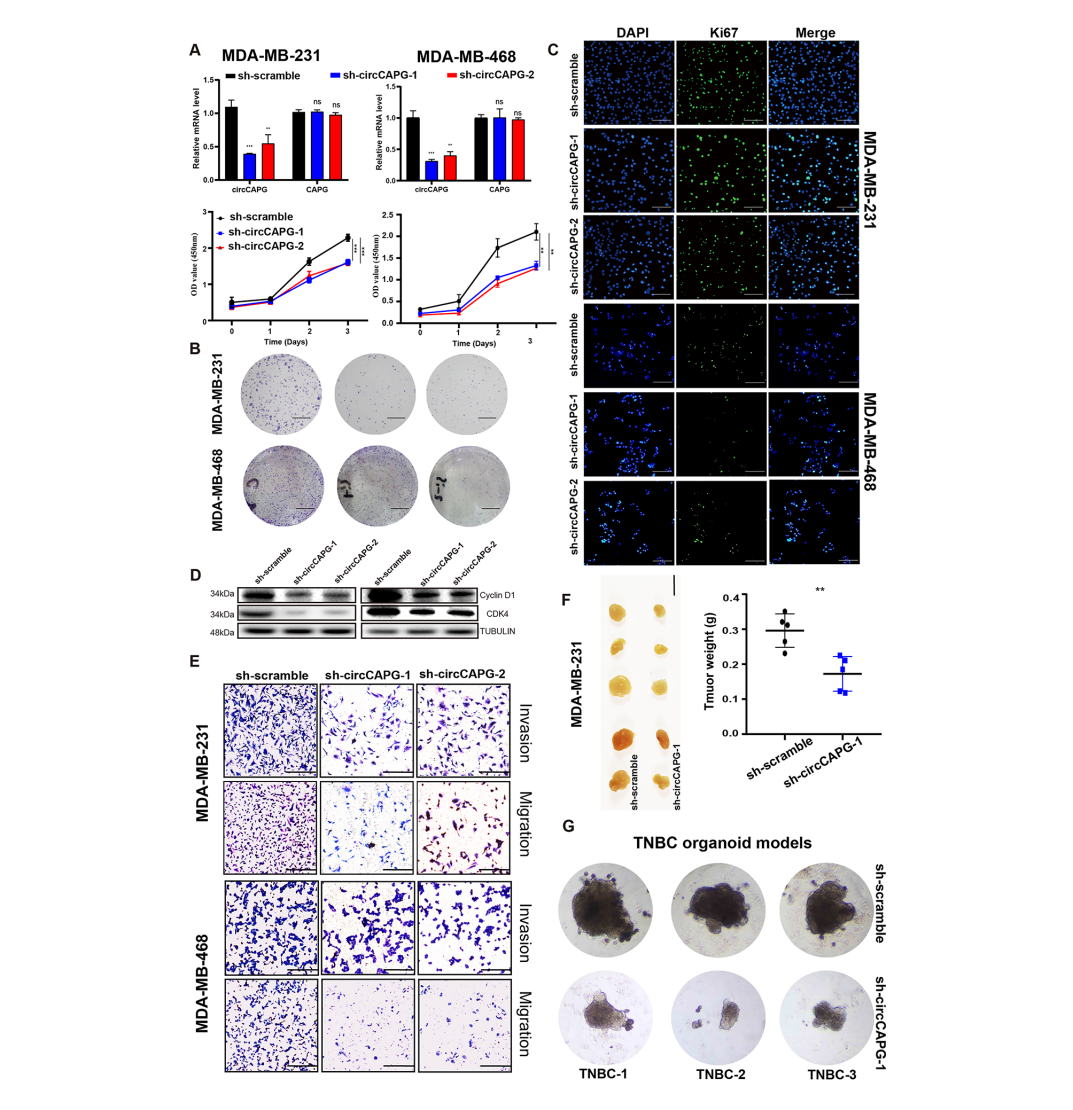
Biological functions of circCAPG in TNBC. **(A)** qPCR of circCAPG and linear CAPG in circCAPG knockdown (KD) MDA-MB-231 and MDA-MB-468. The cell proliferation was estimated by OD450 value of circCAPG KD MDA-MB-231 and MDA-MB-468. **(B)** Colony formation assay of circCAPG KD in MDA-MB-231 and MDA-MB-468 (scale bars, 1 cm). **(C)** Representative immunofluorescence images of circCAPG KD MDA-MB-231 and MDA-MB-468 stained with Ki67 antibody (scale bars, 50 μm). **(D)** Immunoblot (IB) of Cyclin D1 and CDK4 in circCAPG KD MDA-MB-231 and MDA-MB-468. Anti-TUBULIN was used as a sample loading control. **(E)** Transwell assay in circCAPG KD MDA-MB-231 and MDA-MB-468 to estimate cell migration and invasion capacity (scale bars,50 μm). **(F)** BALB/c nude mice (n = 5 for each group) were injected with sh-circCAPG-1 or sh-scramble MDA-MB-231 cells (scale bars, 1 cm). The weights of xenograft tumors were summarized after animals were sacrificed. **(G)** Image of TNBC patient-derived organoids infected with lentivirus encoding control or circCAPG shRNAs. All data were representative of at least three biological replicates and shown as mean ± SEM. *P < 0.05, **P < 0.01, ***P < 0.001

To explore the potential biological functions of circCAPG in TNBC, three siRNAs were designed precisely to target the back-splicing junction sites of circCAPG without affecting the linear transcription of CAPG (Fig. S2A). We found that circCAPG depletion by short hairpin RNAs (shRNAs) has no significant effects on the expression levels of both mRNA and protein of CAPG (Fig. 2A, Fig. S2B). Cell viability, colony formation and immunofluorescence assays presented significantly inhibited proliferative ability (Fig. 2A-C) of TNBC cells by circCAPG KD. The results of Western blotting showed that the cell cycle was arrested (Fig. 2D). However, circCAPG KD did not induce apoptosis (Fig. S2C). In addition, the silence of circCAPG can suppress both migration and invasion of TNBC cells (Fig. 2E). In parallel with the in vitro results, TNBC-bearing xenograft model showed a significant decrease in both volumes and weights of tumors in sh-circCAPG groups compared with the controls (Fig. 2F). To further explore the clinical therapeutic effect of circCAPG, we prepared a TNBC organoid models from three patients. Results showed that circCAPG KD significantly inhibited the growth of TNBC organoids (Fig. 2G). Taken together, circCAPG KD could suppress the proliferation and metastasis of TNBC.

### A novel polypeptide CAPG-171aa was encoded by circCAPG

**Fig. 3:**
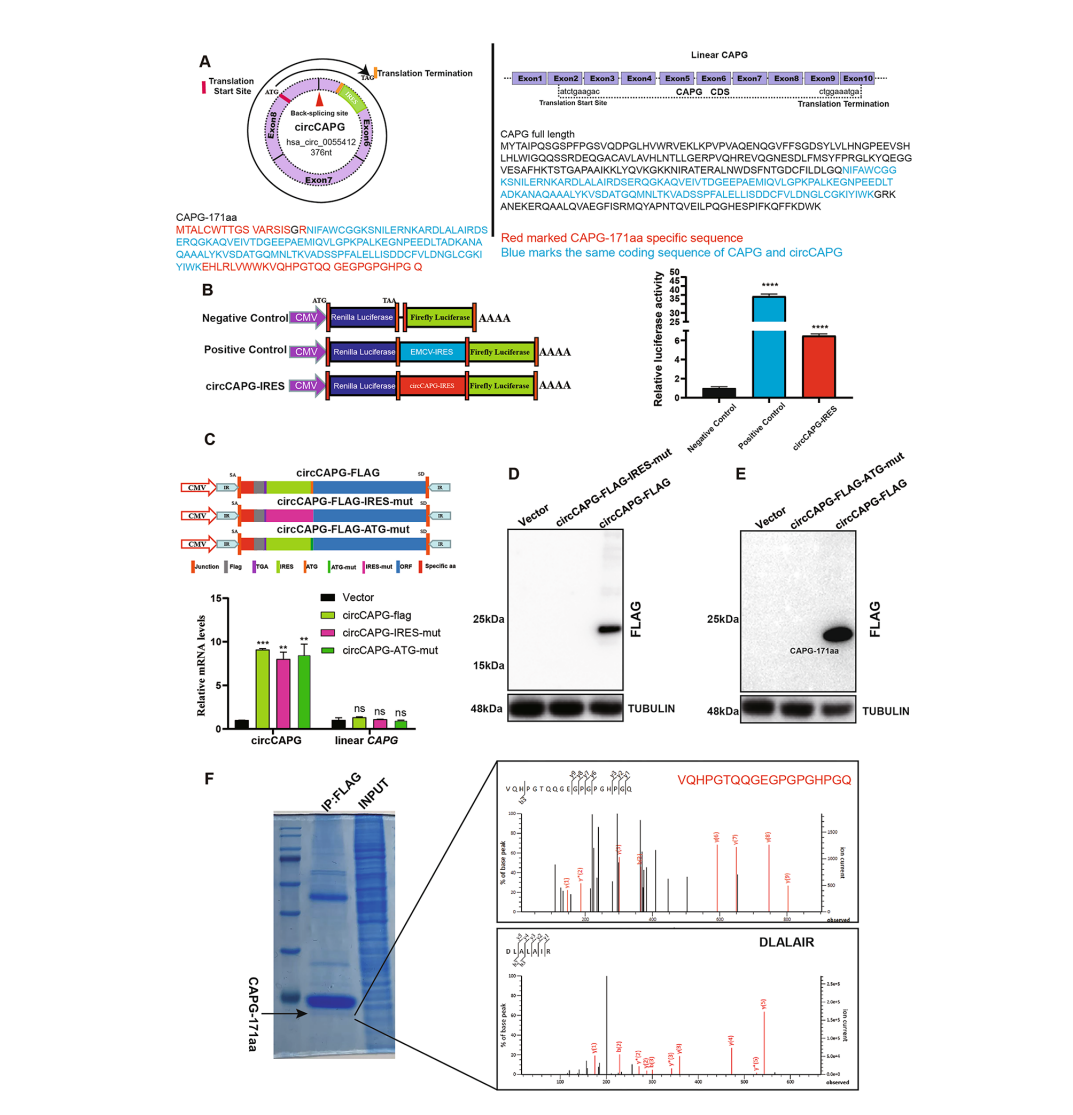
CircCAPG encodes a novel protein named CAPG-171aa. **(A)** An open reading frame (ORF) of circCAPG and its start and end codon were labeled (Left). All exons and ORF of CAPG and its start and end codon were labeled (Right). Red marked CAPG-171aa specific amino acid sequence. Blue marked the same coding sequence of CAPG and circCAPG. **(B)** Three modified dual-luciferase vectors, luciferase-fired, EMCV-IRES-inserted, and circCAPG-IRES-inserted vectors were established to determine IRES activity of circCAPG. Luciferase-fired and EMCV-inserted vectors work as negative and positive controls, respectively. **(C)** Mutant IRES (circCAPG-FLAG-IRES-mut) and ATG (circCAPG-FLAG-ATG-mut) sequence of circCAPG was designed to validate the coding potential of circCAPG. qPCR of circCAPG and linear CAPG was then tested. **(D)** IB of CAPG-171aa in circCAPG-FLAG and circCAPG-FLAG-IRES-mut cells. **(E)** IB of CAPG-171aa in circCAPG-FLAG and circCAPG-FLAG-ATG-mut transfected 293T. **(F)** IB of CAPG-171aa in circCAPG-FLAG through Immunoprecipitating (IP) FLAG antibody. The right graph shows the amino acid sequences of the junction site of circCAPG determined by mass spectrometry (MS). All error bars represent SEM, two-tailed Student’s t-test, *P < 0.05, **P < 0.01, ***P < 0.001, ns denotes no significance

As we all know, circRNAs can serve as a microRNA (miRNA) “sponge” [16]. We then explored whether circCAPG suppresses TNBC progress by interacting with miRNAs. The anti-argonaute 2 (AGO2) complex RNA immunoprecipitation (RIP) assays are the only mainstream method widely used at present to research sponge function of circRNA [7]. Therefore, we conducted a RIP experiment using AGO2, and the results showed that AGO2 could not accumulate circCAPG (Fig.S3A). Based on the results predicted by the online database circRNADb [17], an open reading frame (ORF) and two internal ribosome entry site sequences (IRES 1, 26–89 sites; IRES 2, 29–86 sites) essential for 5’ cap-independent translation were detected in circCAPG, which suggest its genetic translation potential (Fig. 3A; Fig. S3B). Dual luciferase gene reporter assay validated the translational initiation capacity of the IRES sequences, which exhibit higher firefly luciferase (F-Luc)/Renilla luciferase (R-Luc) activity compared with the negative control (Fig. 3B). We hypothesized this junction-spanning ORF driven by the IRES potentially encoded a 171-amino acid protein (CAPG-171aa). To confirm this prediction that a 171-aa protein was encoded by circCAPG, we designed several circCAPG overexpression plasmids carrying mutated IRES (circCAPG-FLAG-IRES-mut) or mutated ATG sequences (circCAPG-FLAG-ATG-mut) in FLAG-tagged vector containing circCAPG ORF (circCAPG-FLAG) as described in Fig. 3C. RT-qPCR results showed that transfection of these vectors in 293T cells elevated the expression of circCAPG without affecting the expression level of CAPG mRNA (Fig. 3C). A ~ 24kD FLAG-tagged protein was detected by Western blotting (Fig. 3D and E). Meanwhile, either circCAPG-FLAG-IRES-mut or circCAPG-FLAG-ATG-mut vector could abolish the FLAG-tag protein expression (Fig. 3D and E). These results showed that only circCAPG-FLAG could be translated into a polypeptide (Fig. 3D and E) named CAPG-171aa. Since parts of CAPG-171aa (about 171 aa) are originated from CAPG, we used an antibody resistant to CAPG to test CAPG-171aa and found higher CAPG-171aa expression in the circCAPG-FLAG overexpression (OE) group than in the controls, which proves the generation of endogenous CAPG-171aa (Fig. S3C and S3D).

By using liquid chromatography-tandem mass spectrometry (LC-MS), we successfully characterized a specific protein fragment (VQHPGTQQGEGPGPGHPGQ) of CAPG-171aa in the circCAPG-FLAG transfected 293T cells (Fig. 3F; Fig. S3E). All the results demonstrated that circCAPG can be translated into a novel polypeptide, CAPG-171aa, in an IRES-dependent manner.

### CAPG-171aa promotes the proliferation and metastasis of TNBC

**Fig. 4:**
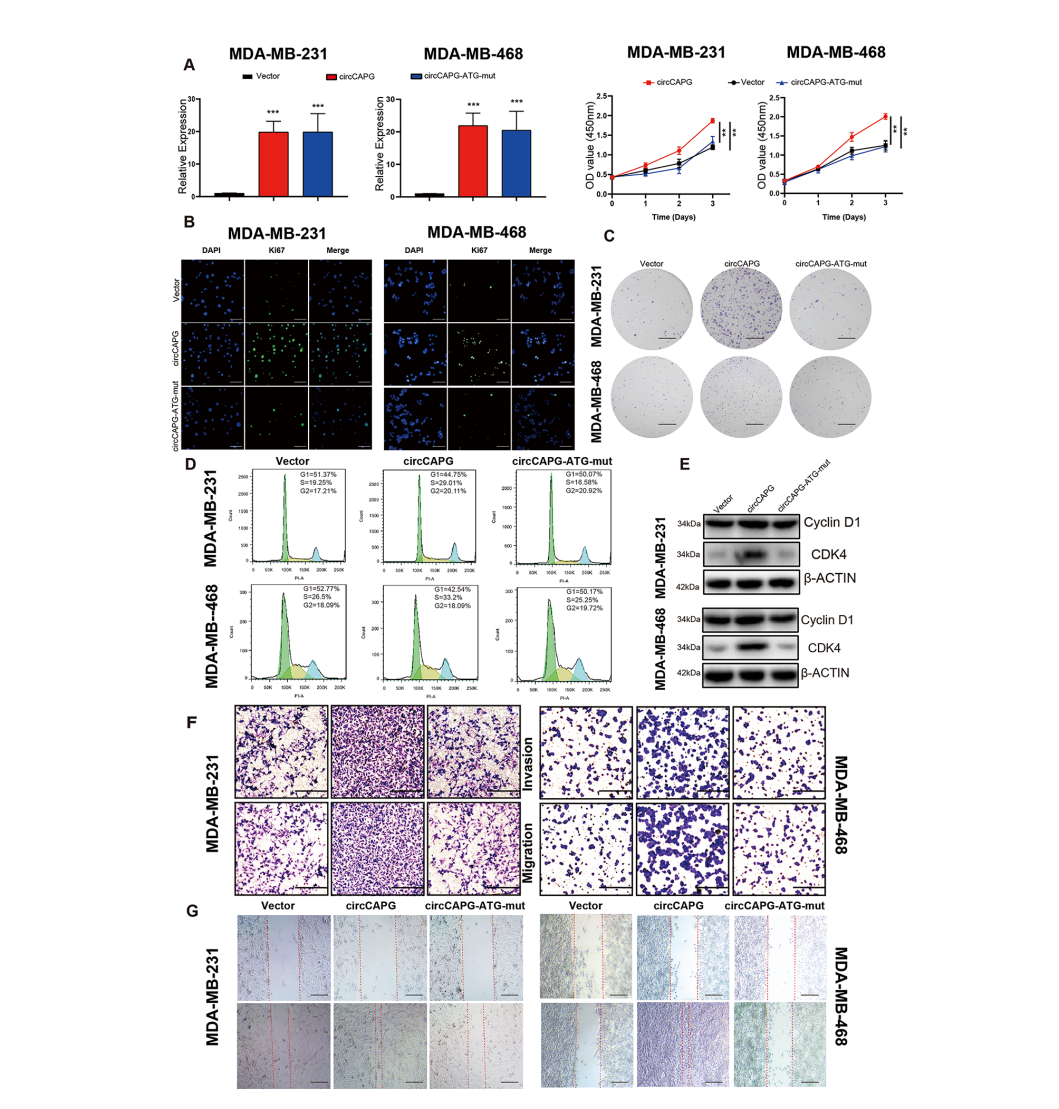
Biological functions of CAPG-171aa in TNBC. **(A)** qPCR of circCAPG and cell proliferation in OE-circCAPG and OE-circCAPG-ATG-mut transfected MDA-MB-231 and MDA-MB-468. **(B)** Ki67 immunofluorescence in OE-circCAPG and OE-circCAPG-ATG-mut transfected MDA-MB-231 and MDA-MB-468 (scale bars, 50 μm). **(C)** Colony formation capacity of OE-circCAPG and OE-circCAPG-ATG-mut transfected MDA-MB-231 and MDA-MB-468 (scale bars, 1 cm). **(D)** Cell cycle assay of OE-circCAPG and OE-circCAPG-ATG-mut transfected MDA-MB-231 and MDA-MB-468 based on flow cytometry. **(E)** IB of cell cycle biomarkers, cyclin D1 and CDK4 in OE-circCAPG and OE-circCAPG-ATG-mut transfected MDA-MB-231 and MDA-MB-468. **(F)** Transwell assay of OE-circCAPG and OE-circCAPG-ATG-mut transfected MDA-MB-231 and MDA-MB-468 (scale bars, 50 μm). **(G)** Wound healing assay of OE-circCAPG and OE-circCAPG-ATG-mut transfected MDA-MB-231 and MDA-MB-468 (scale bars,50 μm). All data were representative of at least three biological replicates and shown as mean ± SEM. *P < 0.05, **P < 0.01, ***P < 0.001

To further explore the biological functions of CAPG-171aa, we generated several TNBC cell lines stably transfected with circCAPG-FLAG, circCAPG-FLAG-ATG-mut or control empty vector (Fig. 3C). The efficiency of circCAPG OE was further verified by performing Western blotting and RT-qPCR (Fig. 4A; Fig. S4A). The cell viability and colony formation assays displayed significantly increased proliferation in TNBC cells under circCAPG OE (Fig. 4B and C), while the circCAPG-FLAG-ATG-mut treatment failed to influence the growth of cells (Fig. 4B and C). Cell cycle was also increased proven by western blotting and flow cytometry in circCAPG OE TNBC cells (Fig. 4D and E). Regarding the mobility of TNBC cells, circCAPG OE increased both migratory and invasive abilities of TNBC cells, whereas circCAPG-FLAG-ATG-mut shown few differences from the control group (Fig. 4F and G). In addition, no significant changes in apoptosis were observed among different groups (Fig. S4B).

Subsequently, to further verify the specific function of CAPG-171aa, we rescued CAPG-171aa expression in circCAPG KD TNBC cell lines by transfecting plasmids containing circCAPG or linearized CAPG-171aa (PLV-CAPG-171aa). Functionally, either re-expression of circCAPG or CAPG-171aa could recover the cell proliferation, migration and invasion abilities in circCAPG KD cell lines (Fig. S4D and F), which suggests that CAPG-171aa could prompt these pathological processes independently. To further detect whether this polypeptide CAPG-171aa plays a role in promoting the growth of TNBC, we subcutaneously implanted stably expressed OE-CAPG-171aa MDA-MB-231 cell line into BALB/c nude mice, and the results showed that OE-CAPG-171aa promotes tumor growth (Fig. S4G and H). Meanwhile, we also stably overexpressed polypeptide CAPG-171aa in the normal breast cell lines MCF-10 A, but no obvious progression of malignancy was observed (Fig. S4I and J).

Based on our results from both in vitro and in vivo, we demonstrated that polypeptide CAPG-171 aa has the great potential to promote the proliferation and metastasis of TNBC cells.

### The binding of CAPG-171aa to STK38 inhibits the proliferation, migration and invasion of TNBC

**Fig. 5:**
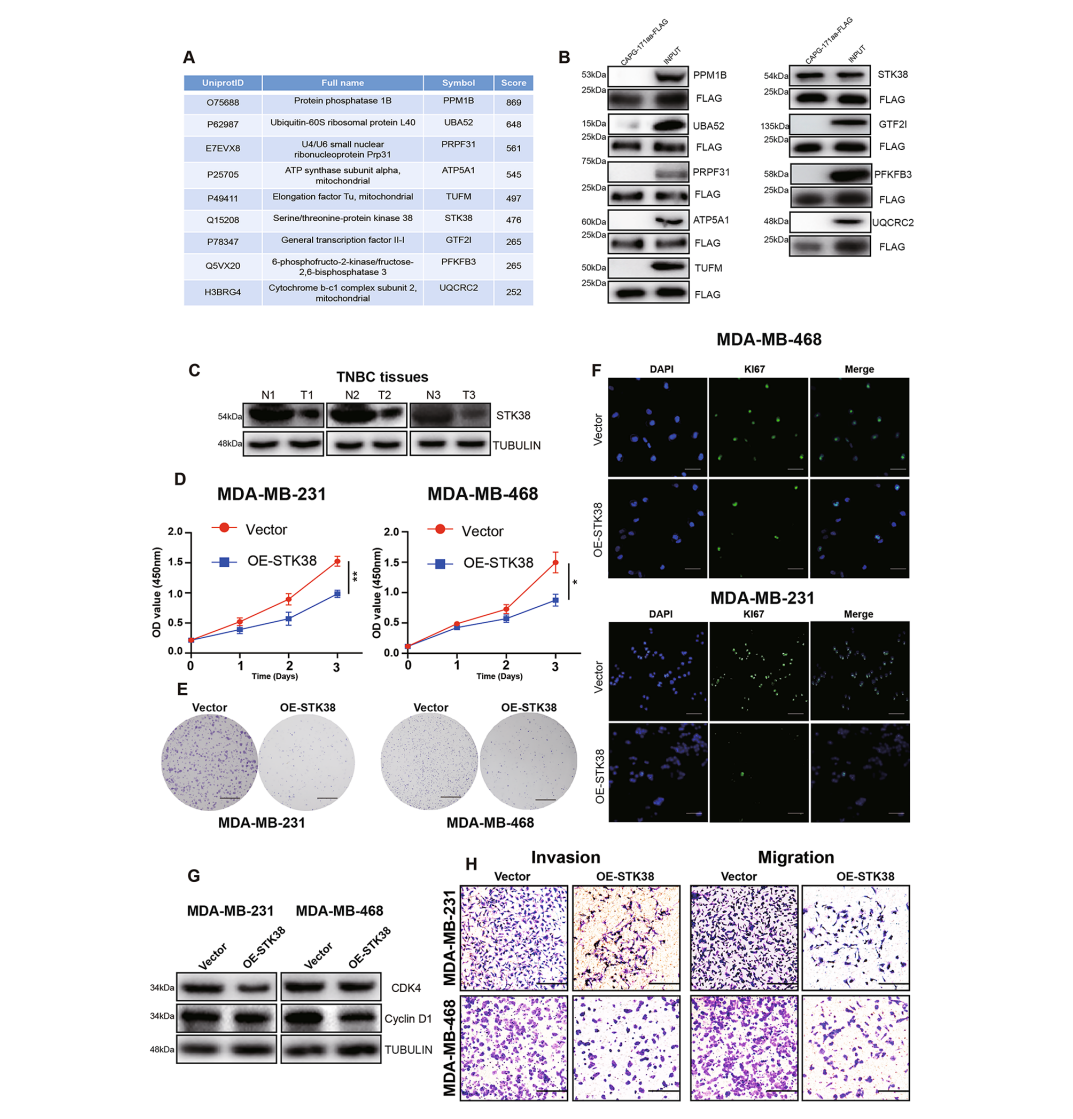
STK38, the interacting protein of CAPG-171aa, promotes the proliferation, migration, and invasion capacity of TNBC. **(A)** Nine interacting proteins of circCAPG were selected through MS and listed with their corresponding scores. **(B)** IB of these nine potential interacting proteins of CAPG-171aa through Co-Immunoprecipitation assay with flag antibody in TNBC cells. **(C)** Western blotting analysis of STK38 in TNBC and its corresponding adjacent normal tissue. **(D)** Cell proliferation in STK38 overexpressed (OE-STK38) MDA-MB-231 and MDA-MB-468 cells. **(E)** Colony formation assay in OE-STK38 transfected MDA-MB-231 and MDA-MB-468 cells. **(F)** Immunofluorescence of GFP in OE-STK38 transfected MDA-MB-231 and MDA-MB-468 cells. **(G)** Western blotting analysis of CDK4 and Cyclin D1 in OE-STK38 transfected MDA-MB-231 and MDA-MB-468 cells. **(H)** Transwell assay in OE-STK38 transfected MDA-MB-231 and MDA-MB-468 to test the migration and invasion ability of cancer cells. All data were representative of at least three biological replicates and shown as mean ± SEM. *P < 0.05, **P < 0.01, ***P < 0.001

To understand the underlying molecular mechanisms by which CAPG-171aa regulates TNBC progression, we performed Immunoprecipitation (IP) in circCAPG-flag overexpressed 293T cells to identify its potential targeted molecules. Mass spectrometry (MS) was utilized to identify the interacting proteins of CAPG-171aa and a total of nine candidate proteins draw our attention (Fig. 5A; Fig. S5A). Based on the results of Co-IP, only STK38 out of these 9 proteins could bind to CAPG-171aa in TNBC cells (Fig. 5B). Furthermore, STK38 did not interact with maternal CAPG (Fig. S5B). STK38 is a member of NDR kinase family [18] and plays a crucial role in tumorigenesis as a tumor suppressor [19] or oncogene [20, 21]. In our study, STK38 protein was found to be lower expressed in TNBC cell lines (Fig. S5C) and tissues of patients (Fig. 5C). The overexpression of STK38 (Fig. S5E) could significantly inhibit cell proliferation (Fig. 5D and F), interrupt cell cycle progression (Fig. 5G), migratory and invasive abilities in TNBC cell lines (Fig. 5H), but did not alter apoptosis (Fig. S5D). Taken together, our results indicated that STK38 limits the proliferation, migration and invasion of TNBC cells.

### CAPG-171aa interacts with STK38 and activates the downstream MEK1/2-ERK1/2 pathway via MEKK2

**Fig. 6:**
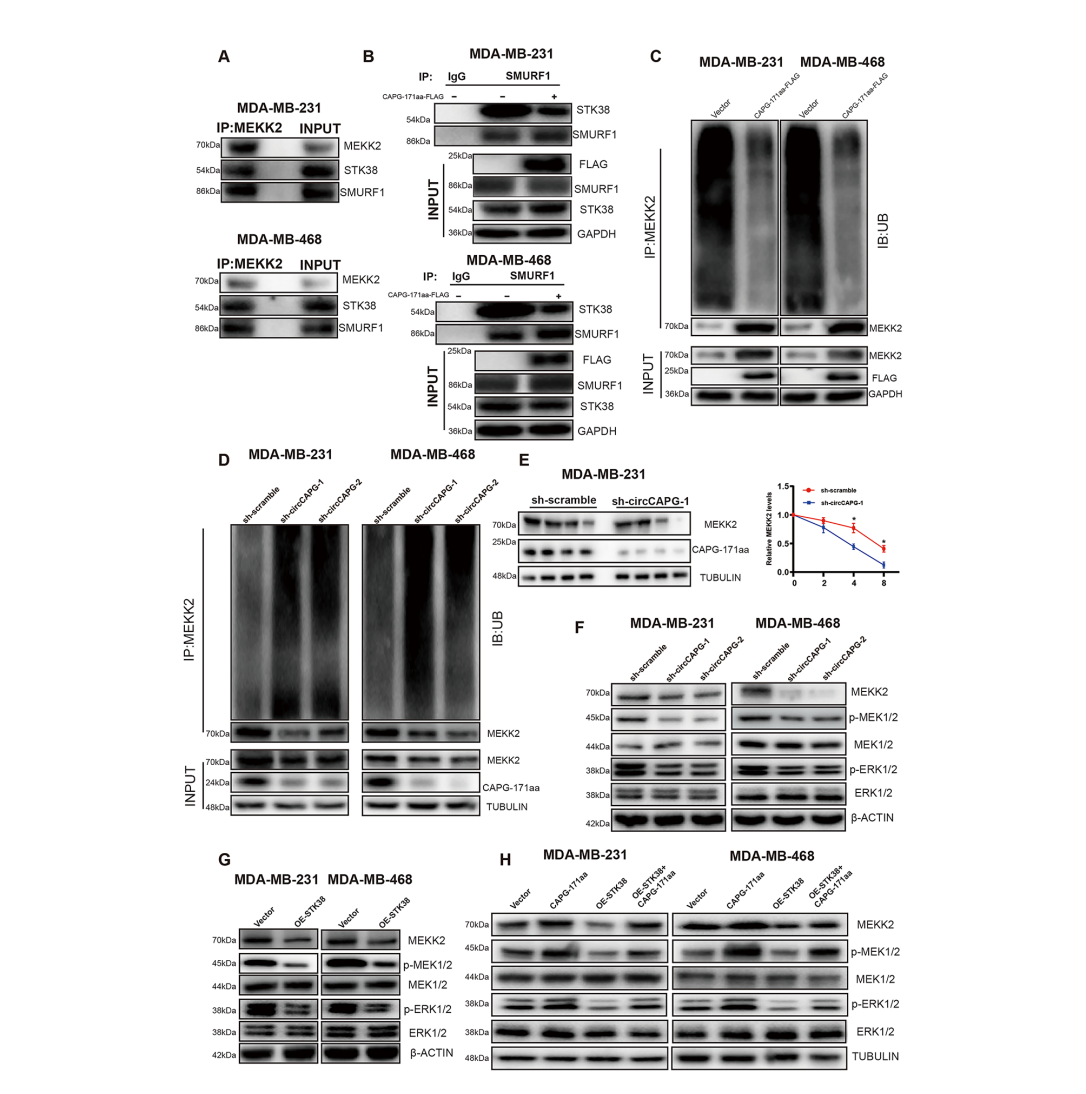
CAPG-171aa interacts with STK38 activating the downstream MEK1/2-ERK1/2 pathway via MEKK2. **(A)** MDA-MB-231 and MDA-MB-468 cell lysates were IP with anti-MEKK2 antibody followed by detection with anti-MEKK2, STK38, and SMURF1 antibody. **(B)** MDA-MB-231 and MDA-MB-468 were transfected with CAPG-171aa-FLAG. Whole-cell lysates were IP with anti-SMURF1 and IgG antibodies followed by detection with anti-FLAG, STK38, SMURF1, and GAPDH antibodies. (C-D) Before being treated with MG132, MDA-MB-231, and MDA-MB-468 were transfected with CAPG-171aa-FLAG and circCAPG KD plasmids. Ubiquitination and protein expression levels of MEKK2 were assayed in CAPG-171aa OE **(C)** and circCAPG KD **(D)** MDA-MB-231 and MDA-MB-468. **(E)** Ubiquitination and protein expression levels of MEKK2 were assayed in circCAPG KD MDA-MB-231 and MDA-MB-468 through pulse-chase experiments with cycloheximide. **(F)** IB of MEKK2, p-MEK1/2, MEK1/2, p-ERK1/2 and ERK1/2 in circCAPG KD MDA-MB-231 and MDA-MB-468. **(G)** IB of p-MEK1/2, MEK1/2, p-ERK1/2 and ERK1/2 in OE STK38 MDA-MB-231 and MDA-MB-468. **(H)** IB of MEKK2, p-MEK1/2, MEK1/2, p-ERK1/2 and ERK1/2 in CAPG-171aa, OE-STK38 and OE-STK38/CAPG-171aa transfected MDA-MB-231 and MDA-MB-468. All data were representative of at least three biological replicates and shown as mean ± SEM. *P < 0.05, **P < 0.01, ***P < 0.001

Previous studies have shown that STK38 can regulate the stability of MYC protein in a kinase-dependent manner [20] and promote the ubiquitination and degradation of mitogen-activated protein kinase kinase 2 (MEKK2) through binding to the SMAD Specific E3 Ubiquitin Protein Ligase 1 (SMURF1) [21]. In our study, although MYC was not affected in TNBC cells (Fig. S6A), silencing circCAPG caused a decrease in the protein level of MEKK2 in TNBC cell lines but not in the transcriptional level (Fig. S6A-B), which indicated that CAPG-171aa might regulate the protein stability of MEKK2. By using IP assays with anti-MEKK2 antibodies, we found that MEKK2 could interact with STK38 and SMURF1 in TNBC cells (Fig. 6A). The further study proved that OE-CAPG-171aa reduced the interaction between STK38 and SMURF1 (Fig. 6B), whereas the expressions of their mRNA and protein were not altered by knockdown circCAPG (Fig. S6C-F). In addition, OE-CAPG-171aa reduced the ubiquitination of MEKK2 and increased MEKK2 protein level (Fig. 6C). Correspondingly, the ubiquitin signals of MEKK2 were increased in circCAPG KD TNBC cell lines (Fig. 6D). Next, we performed a pulse-chase experiment with cycloheximide (CHX) to inhibit the de novo synthesis of MEKK2 proteins. A decreased protein level of MEKK2 was found in the circCAPG-silenced group (Fig. 6E; Fig. S6G). Because of the important role of MEKK2 in tumorigenesis and metastasis through phosphorylating the downstream MAPK kinase 1/2 (MEK1/2) and extracellular regulated kinase 1/2 (ERK1/2) [21], we analyzed the proteins expressions of the MEKK2-MEK1/2-ERK1/2 with circCAPG-silenced and STK38 OE TNBC cell lines. Either silenced circCAPG or OE STK38 could attenuate the phosphorylation of MEK1/2 and ERK1/2 in TNBC cells (Fig. 6F and G). In contrast, the decreased phosphorylation of MEK1/2 and ERK1/2 in STK38 OE TNBC cells was able to be rescued by overexpressing CAPG-171aa (Fig. 6H).

In addition, we silenced MEKK2 in CAPG-171aa OE TNBC cell lines. The siRNAs were designed precisely to target the MEKK2 and transfected into CAPG-171aa OE TNBC cell lines. Functionally, MEKK2 KD could reduce cell proliferation (Fig.S6H and 6I), migration and invasion abilities (Fig. S6J) in CAPG-171aa OE TNBC cell lines, which suggests that MEKK2 is the downstream partner of CAPG-171aa.

All the above results indicated that the interaction between CAPG-171aa and STK38 could inhibit the ubiquitination of MEKK2 protein and then activate the MEK1/2-ERK1/2 pathway.

### The formation of circCAPG is regulated by ***SLU7*** through binding to the flanking intronic complementary sequences of the CAPG pre-mRNA

**Fig. 7:**
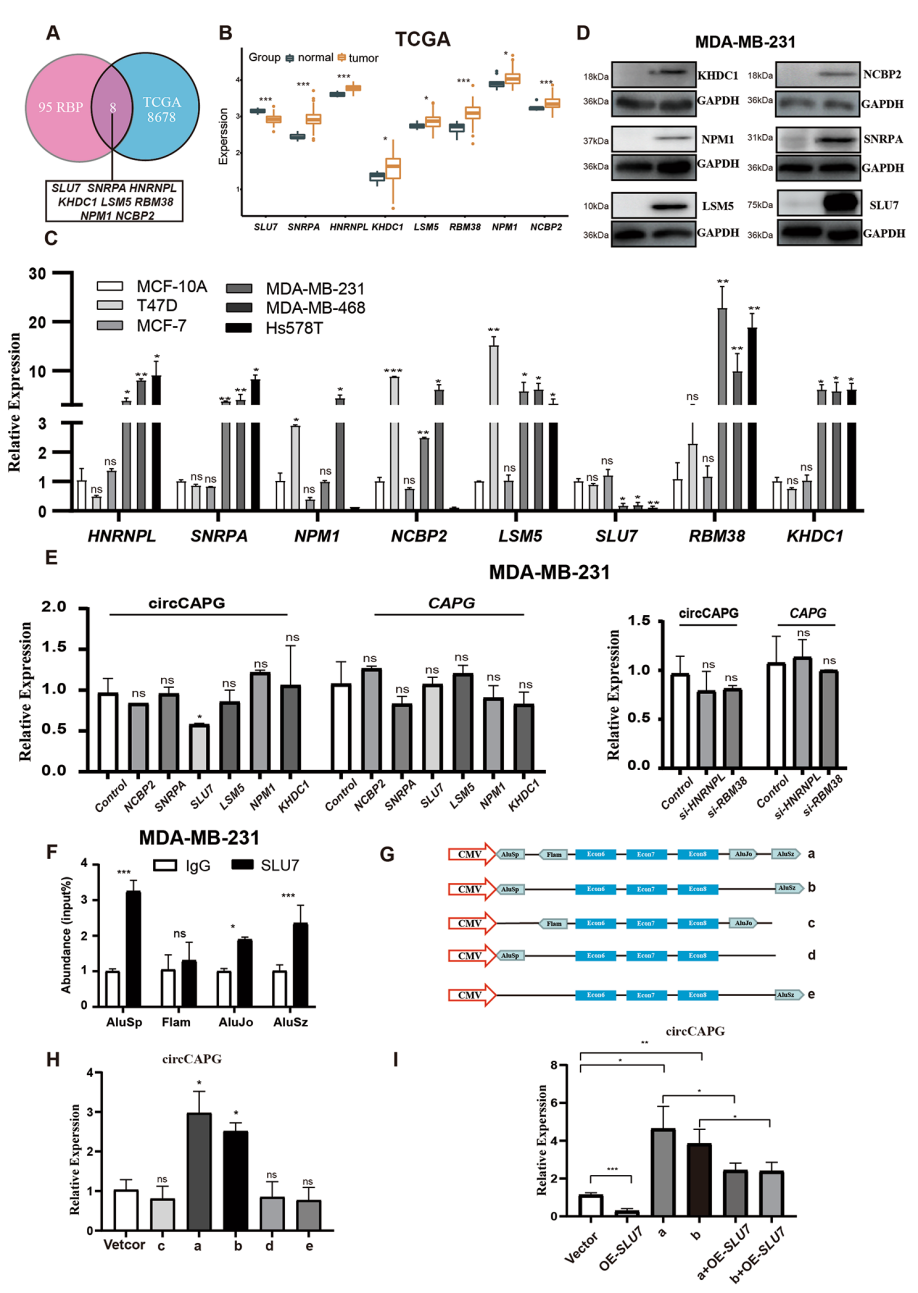
SLU7 regulated the generation of circCAPG. **(A)** Eight RBPs were selected based on both the previous studies and the TCGA database. **(B)** The expression level of these RBPs in both tumor and normal tissue. **(C)** The relative expression level of these eight RBPs in breast cancer cell lines. **(D)** IB of eight RBPs in MDA-MB-231. **(E)** qPCR of circCAPG and linear CAPG in RBP OE (left) and KD (right) MDA-MB-231. **(F)** RIP with an anti-SLU7 and IgG antibody in MDA-MB-231 cell lines was used to detect the mRNA levels of flanking intronic sequences (AluSp, Flam, AluJo, and AluSz) of circCAPG. **(G)** Illustration of five constructs designed with different combinations of four Alu sequences randomly inserted before exon 6 and/or after exon 8 of circCAPG. **(H)** qPCR of circCAPG in a, b, c, d, and e constructs. **(I)** qPCR of circCAPG in a, b, a + OE-Slu7, and b + OE-Slu7. All data were representative of at least three biological replicates and shown as mean ± SEM. *P < 0.05, **P < 0.01, ***P < 0.001

To clarify the molecular mechanisms of circCAPG formation in TNBC, we searched the CircInteractome database to predict the potential RBP sites in the flanking regions of circCAPG. The preliminary prediction with CircInteractome (https://circinteractome.nia.nih.gov/index.html) [22] exported several eukaryotic translation initiation factor 4A3 (*EIF4A3*) binding sites to circCAPG, which located in the upstream and downstream regions of circCAPG transcripts (Fig. S7A), but *EIF4A3* KD did not affect the transcriptional level of circCAPG (Fig. S7B). Given the previous studies reporting 103 of RBPs involved in the biogenesis of circRNA [11], we examined the expression levels of these RBPs in both TNBC tissues from the cancer genome atlas database (TCGA). Finally, a total of eight RBPs were observed to be dysregulated (Fig. 7A and B) and we confirmed this differential expression in different breast cancer cell lines as well (Fig. 7C). Subsequently, overexpression or knockdown of these eight RBPs was conducted in TNBC cell lines. Based on our results, only *SLU7* can lead to decreased circCAPG expression without affecting linear CAPG meanwhile at the transcriptional level (Fig. 7D and E; Fig. S7C and S7D). Thus, we focused on *SLU7* in the generation of circCAPG in TNBC. It has been reported that *SLU7* (an important splicing factor) can bind to the proximal AG of the Alu sequence to regulate the splicing process of pre-mRNA [23]. Therefore, we examined the flanking intronic regions of circCAPG and four potential *SLU7* binding sites (AluSp and AluSz, AluJo and Flam) were found (Fig. S7E). The interactions between *SLU7* and these binding sites on circCAPG were validated by RIP-qPCR experiments, and the results showed that both the upstream and downstream flanking intronic regions of circCAPG (AluSp, AluSz, and AluJo) were significantly enriched by *SLU7* protein (Fig. 7F; Fig. S7F). Considering the studies ICSs are important for the generation of circRNAs [16] and the high homology between AluSp and AluSz (Fig. S7G), we tested the formation rate of circCAPG by introducing either one or two Alu sequences to the upstream of exon 6 and/or downstream of exon 8 of circCAPG with self-designed plasmids (Fig. 7G). The RT-qPCR results exhibited that both AluSp and AluSz are essential for the formation of circCAPG, while AluJo is dispensable (Fig. 7H). To further investigate the SLU7-Alu mediated circCAPG formation, we simultaneously overexpressed *SLU7* and the constructs carrying different binding sites of *SLU7* (AluSp, AluSz, or Alujo) in MDA-MB-231 cells. The results demonstrated that *SLU7* OE significantly reduced the expression of circCAPG in the cells carrying both AluSp and AluSz (Fig. 7I). These results indicate that the formation of circCAPG requires the binding between SLU7 and ICS (AluSp and AluSz).

### The essential role of ***SLU7***in TNBC cell proliferation, migration and invasion

**Fig. 8:**
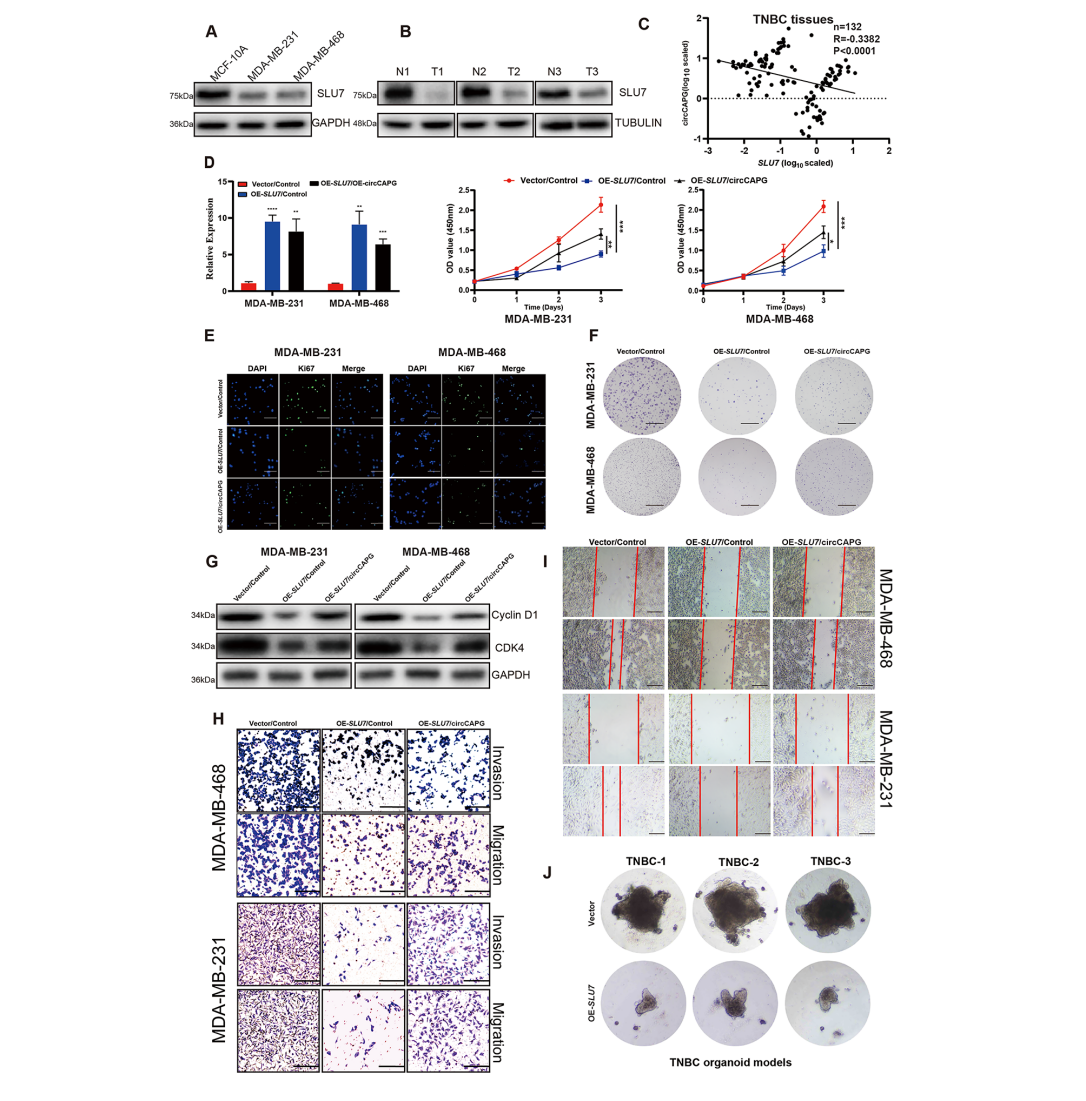
The critical role of SLU7 in TNBC. **(A)** IB of SLU7 in MCF-10 A, MDA-MB-231, and MDA-MB-468. **(B)** IB of SLU7 in TNBC and normal tissue. **(C)** The correlation analysis between SLU7 and circCAPG in TNBC tissue. n = 132. **(D)** Cell proliferation assay of TNBC stable cell lines expressing control vector (Vector), SLU7 followed with infection of indicated control or circCAPG (OE-circCAPG). (scale bars, 1 cm). **(E)** Immunofluorescence with KI67 antibody in TNBC stable cell lines expressing control vector (Vector), SLU7 followed with infection of indicated control or circCAPG (scale bars, 50 μm). **(F)** Colony formation assay in TNBC stable cell lines expressing control vector (Vector), SLU7 followed with infection of indicated control or circCAPG. (scale bars, 1 cm) **(G)** IB of Cyclin D1 and CDK4 in TNBC stable cell lines expressing control vector (Vector), SLU7 followed with infection of indicated control or circCAPG. **(H)** Transwell assay of TNBC stable cell lines expressing control vector (Vector), SLU7 followed with infection of indicated control or circCAPG. (scale bars, 50 μm). **(I)** Wound healing assay of TNBC stable cell lines expressing control vector (Vector), SLU7 followed with infection of indicated control or circCAPG. (scale bars, 50 μm). **(J)** Image of TNBC patient-derived organoids infected with lentivirus expressing control or SLU7. All data were representative of at least three biological replicates and shown as mean ± SEM. *P < 0.05, **P < 0.01, ***P < 0.001

To further evaluate the association between *SLU7* and circCAPG, RT-qPCR was performed in tumors (n = 132) and adjacent tissues of TNBC patients. We found that circCAPG was higher expressed in TNBC tissues. In addition, SLU7 was lower expressed compared with the controls in both TNBC cell lines (Fig. 8A) and three TNBC patients’ tissues (Fig. 8B) by Western blotting. In parallel, the expression of circCAPG was negatively correlated with the expression of SLU7 in TNBC tissues (n = 132) (Fig. 8C). To examine the biological function mediated by *SLU7*-induced downregulation of circCAPG in TNBC, we overexpressed *SLU7* in cells and found that *SLU7* OE significantly inhibited the proliferative ability (Fig. 8D-F) and cell cycle (Fig. 8G), but did not induce apoptosis in TNBC cells (Fig. S7I). Apart from these, the migratory and invasive abilities of TNBC cells were also inhibited by *SLU7* OE (Fig. 8H-I). Moreover, OE-*SLU7* significantly inhibited the growth of TNBC organoids derived from patients (Fig. 8J) and tumor growth in nude mice as well (Fig. S7J and S7K). However, these drastic antitumor phenotypes were partially abolished by circCAPG OE (Fig. 8E-I), which is also consistent with our finding that circCAPG is negatively regulated by *SLU7* (Fig. 8C). This abolishment is not fully complemented, indicating that *SLU7* may also involve in the regulation of other circRNAs, which remains to be studied.

## Discussion

TNBC is a heterogeneous and fatal disease in women with limited treatment options [24]. Therefore, the discovery of a new targeted therapy strategy and biomarker is urgent. Herein, we found a novel circRNA, circCAPG, which was formed by the exons 6–8 of the CAPG gene and has not been studied so far. In the present study, we found that the expression of circCAPG was upregulated in TNBC, and was tightly associated with tumor invasion, metastasis and poor overall survival. We further demonstrated that circCAPG encodes a novel polypeptide named CAPG-171aa driven by an active IRES. In vitro, overexpression studies confirmed the tumor promoting role of CAPG-171aa rather than circCAPG in the progression of TNBC. Mechanism studies revealed that CAPG-171aa inhibited the ubiquitination of MEKK2 by interacting with STK38 and thereby activated the downstream MEK1/2-ERK1/2 signaling pathway. Most importantly, we found that the RNA-binding protein *SLU7* regulates the generation of circCAPG by binding the ICSs of the circRNA’s pre-mRNA. Furthermore, we demonstrated that the expression levels were positively correlated with overall survival of the TNBC in a 10-year follow-up study with 132 patients, the area under the curve of receiver operating characteristic was 0.8723 with 100% specificity and 80% sensitivity. Altogether, results demonstrated that circCAPG might serve as a novel biomarker for diagnosis and prognosis, and a potential therapeutic target for TNBC as well.

The vast majority of circRNA studies were focused on its function as ‘microRNA sponges’ not only in TNBC but also in some other diseases [25, 26], whereas only few studies from our group [7, 14] or others reported its translational function [13, 15]. Therefore, we first explored whether circCAPG regulates TNBC progression through sponge function. However, we noticed that circCAPG barely interacts with miRNAs in sponge manner in TNBC. With the development of biotechnology and online bio-software, circRNAs driven by certain IRES [6, 7] or N6-methyladenosine (m6A) [27] with potential protein-coding functions could be predicted and some have been reported recently. Our previous work also proved the existence of protein-encoded circRNAs [7, 14]. All this prompted us to explore the possibility and the molecular effects of circCAPG-derived translative proteins or polypeptides on the malignant behaviors of TNBC. After evaluating the sequence of circCAPG at the circRNADb website [17], both putative IRES structure and ORF of circCAPG were predicted and obtained, suggesting its potential protein-coding capacity. Since IRES is a key promoter for circRNA translation, a circCAPG-flag-IRES-mut plasmid was constructed to test the expression of CAPG-171aa in comparison with the putative circCAPG-IRES construct. The results demonstrated that the sequence of circCAPG owns the IRES-derived translational initiation capability and polypeptide-coding ability. By a series of experiments, we further confirmed that this circRNA-encoded polypeptide CAPG-171aa rather than circCAPG itself promoted the proliferation and metastasis of TNBC cells. Although our results showed the tumor promoting effect of CAPG-171aa on TNBC in BALB/c nude mice and TNBC cell lines, and its enhancing role in the migration and invasion of TNBC cells in vitro, a serial of suitable TNBC-metastasis models to further confirm whether CAPG-171aa-induced TNBC metastatic outcome and tumorigenesis in vivo was missing in this study. To figure out the molecular action of how CAPG-171aa in TNBC metastasis and tumorigenesis would help better understanding the mechanism of TNBC metastasis, which is also our task and is carried out in our ongoing study. In addition, no observed malignant changes in a polypeptide CAPG-171aa stable overexpressed MCF-10A normal control cell line is still an open question (Fig. S4I and J). The reason for this might be due to the complexity and the context of tumorigenesis of TNBC, that massive oncogenic factors and the micro environment should be co-operated together at the same time to prompt the development of TNBC, rather than a single one can orchestra the carcinogenesis, which is also another task for our future study.

In our study, STK38 was identified as an important binding partner of CAPG-171aa proven by MS analysis and IP experiments. Interestingly, STK38 does not interact with maternal CAPG protein. This further reflects that CAPG-171aa involved in the progression of TNBC as a novel oncoprotein. STK38 is a serine-threonine protein kinase that belongs to a subfamily of the AGC kinase family [18]. It can regulate the protein levels of MYC in a kinase activity-dependent manner [20] and promote SMURF1-mediated polyubiquitination of MEKK2 [21]. Consistent with the study by Ji et al. [21], we found that STK38 inhibits the protein levels of MEKK2 through SMURF1-mediated polyubiquitination and degradation. Most importantly, we found that interaction between CAPG-171aa and STK38 could inhibit the ubiquitination of MEKK2 protein and then activate the MEK1/2-ERK1/2 pathway, which in turn promotes the progression of TNBC.

Notably, investigating the biogenesis mechanisms of circRNAs could help us better understand how circRNAs were generated and accumulated specifically in TNBC progression [28]. However, only a few literatures reported the biogenesis mechanisms of circRNA [27]. Recently, Li et al. reported that ICS and RBP were critical for the formation of circRNAs [11]. Pairing between ICSs at different introns was often thought to be able to bring the distal splice sites closer together and thus promote the biogenesis of circRNA [29–32]. Zheng et al. [32] reported that the inverted repeat Alu elements (IRAlus) of the intron flanking homeodomain interacting protein kinase 3 (HIPK3) Exon2 mediated the formation of circHIPK3 which plays an important role in the etiology and carcinogenesis. Except for ICS, some RNA-binding proteins (RBPs) also engaged in the biogenesis of circRNA. For example, Zeng et al. [33] reported that the splicing factor epithelial splicing regulatory protein 1 (ESRP1) can increase circANKS1B production. However, it is still unclear how RBPs compete or cooperate with the inverted-repeat Alu elements to balance the production of circRNAs. In our study, we found that the expression of circCAPG was negatively correlated with the expression of *SLU7* in TNBC tissues (n = 132). Furthermore, overexpression of *SLU7* can reduce the expression of circCAPG. To examine the SLU7-induced downregulation of circCAPG in TNBC, we carried out a series of experiments and found that the formation of circCAPG requires the binding between *SLU7* and ICS (AluSp and AluSz). In addition, our in vitro studies further demonstrated that *SLU7* OE suppressed the malignant progression of TNBC, while the subsequent supplement of circCAPG OE could not fully promote the malignant phenotype of TNBC. One RBP can regulate many circRNAs, which means *SLU7* could also interfere with the expressions of many other circrRNAs except for circCAPG. For example, the knockdown of *HNRNPL* can lead to 139 significantly upregulated and 93 downregulated circRNAs [34]. The issue that we have not fully addressed is that although *SLU7* regulates the formation of circCAPG in TNBC, the cognate mRNA of the circRNA seems to be unimpacted, which is inconsistent with the previous research [35]. This might be related to the special role of circCAPG in TNBC. Further studies are required to fully elucidate the transcriptional regulation as well as the transcriptional output on circRNAs.

By Kaplan-Meier survival analysis, we found that TNBC patients who had higher circCAPG expression also showed worse prognosis. The receiver operating characteristic (ROC) curve of circCAPG showed that circCAPG yields an excellent diagnostic ability. This suggests that circCAPG has the potential to work as a biomarker in TNBC patients. Unfortunately, we did not get a clear positive band of CAPG-171aa in the serum of TNBC patients by Western blotting with anti-CAPG antibody. One reason for this failure might be due to the lack of a CAPG-171aa-specific antibody. To increase the detection specificity and sensitivity for CAPG-171aa, we are going to make the antibody for ELISA assay in our future study. We do believe that CAPG-17aa determination in a larger scale in TNBC patients would be necessary and enable our better understanding for its diagnostic value.

## Conclusions

In conclusion, circCAPG can significantly enhance the proliferation and metastasis of TNBC cells by encoding a novel polypeptide CAPG-171aa and activating the MEKK2-MEK1/2-ERK1/2 pathway. Additionally, the formation of circCAPG is found to be mediated by *SLU7*. Briefly, the newly discovered encoding circRNA, circCAPG, broadens our knowledge of the underlying tumor promoting mechanisms of circRNAs in TNBC and is able to serve as a novel prognostic biomarker candidate or therapeutic target for the future treatment of TNBC patients.

## Electronic supplementary material

Below is the link to the electronic supplementary material.

Supplementary Material 1

Supplementary Material 2

Supplementary Material 3

Supplementary Material 4

## Data Availability

The datasets used and analyzed during the current study are available from the corresponding author upon reasonable request.

## Abbreviations

circRNA: Circular RNA
TNBC: Triple-negative breast cancer
hsa_circ_0055412: CircCAPG
KD: Knockdown
STK38: Serine/threonine kinase 38
SMURF1: SMAD-specific E3 ubiquitin protein ligase 1
SLU7: SLU7 Homolog- Splicing Factor.
MEKK2: Mitogen-activated protein kinase kinase 2
Co-IP: Coimmunoprecipitation
DAPI: 4’, 6-diamidino-2-phenylindole
GAPDH: Glyceraldehyde-3-phosphate dehydrogenase
gDNA: Genomic DNA
IRES: Ribosome entry site
ORF: Open reading frame
RT-PCR: Reverse transcriptase PCR
ACTD: Actinomycin D
AGO2: Argonaute 2
RIP: RNA immunoprecipitation
