# Supplementary material for: A novel polypeptide CAPG-171aa encoded by circCAPG plays a critical role in triple-negative breast cancer
Source: Mol Cancer. 2023 Jul 5;22:104. doi: 10.1186/s12943-023-01806-x (PMC10320902; doi:10.1186/s12943-023-01806-x)

Fig. 1B

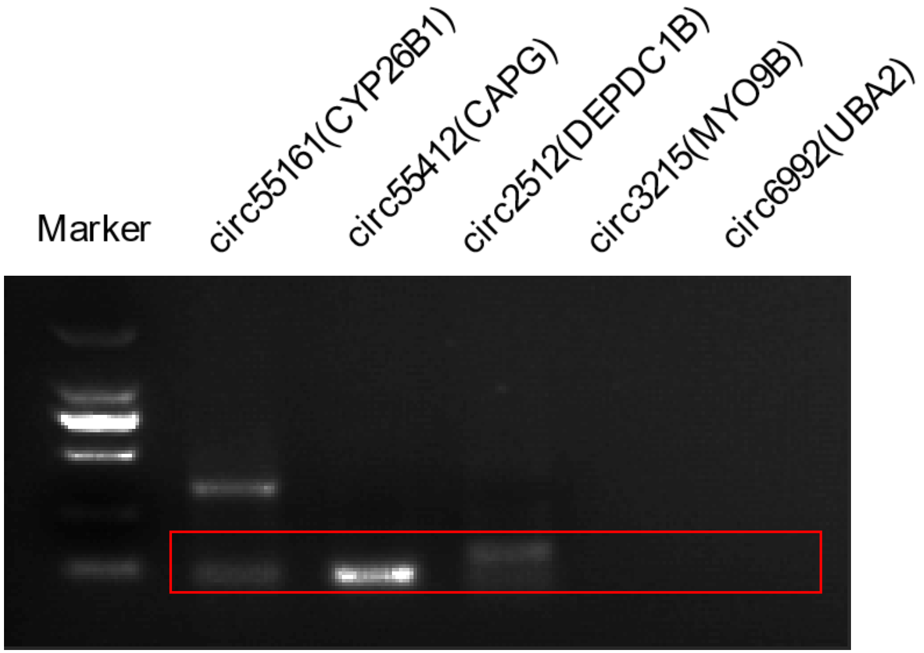
Marker
circ55161(CYP26B1)
circ55412(CAPG)
circ2512(DEPDC1B)
circ3215(MYO9B)
circ6992(UBA2)

Fig. 1F

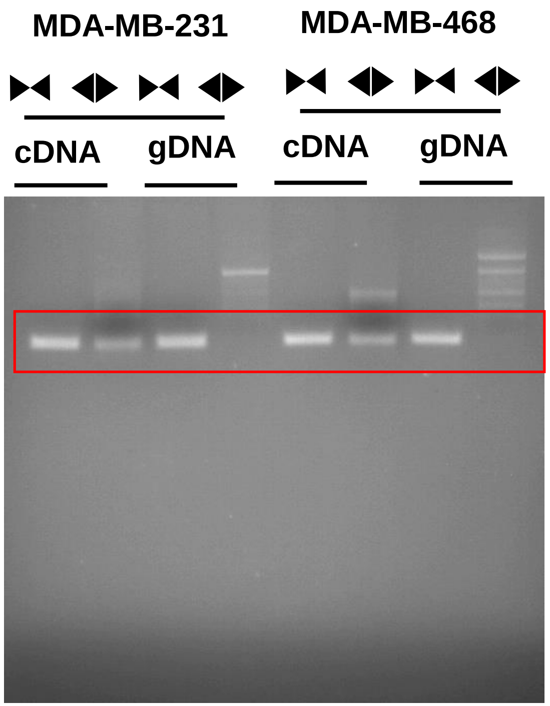
MDA-MB-231
MDA-MB-468
cDNA
gDNA
cDNA
gDNA

Fig. 2D

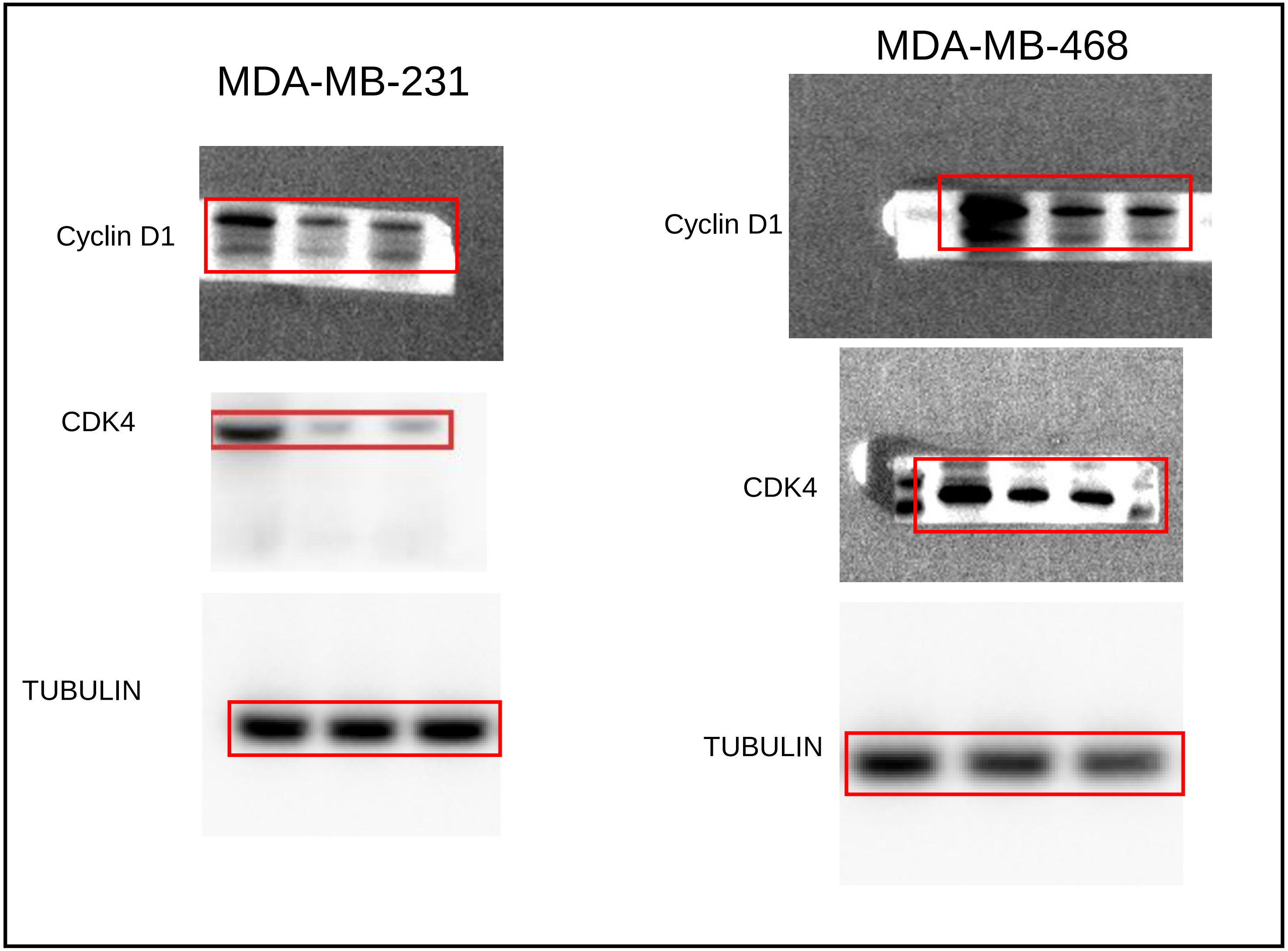
MDA-MB-231
MDA-MB-468
Cyclin D1
CDK4
TUBULIN
Cyclin D1
CDK4
TUBULIN

Fig. 3D

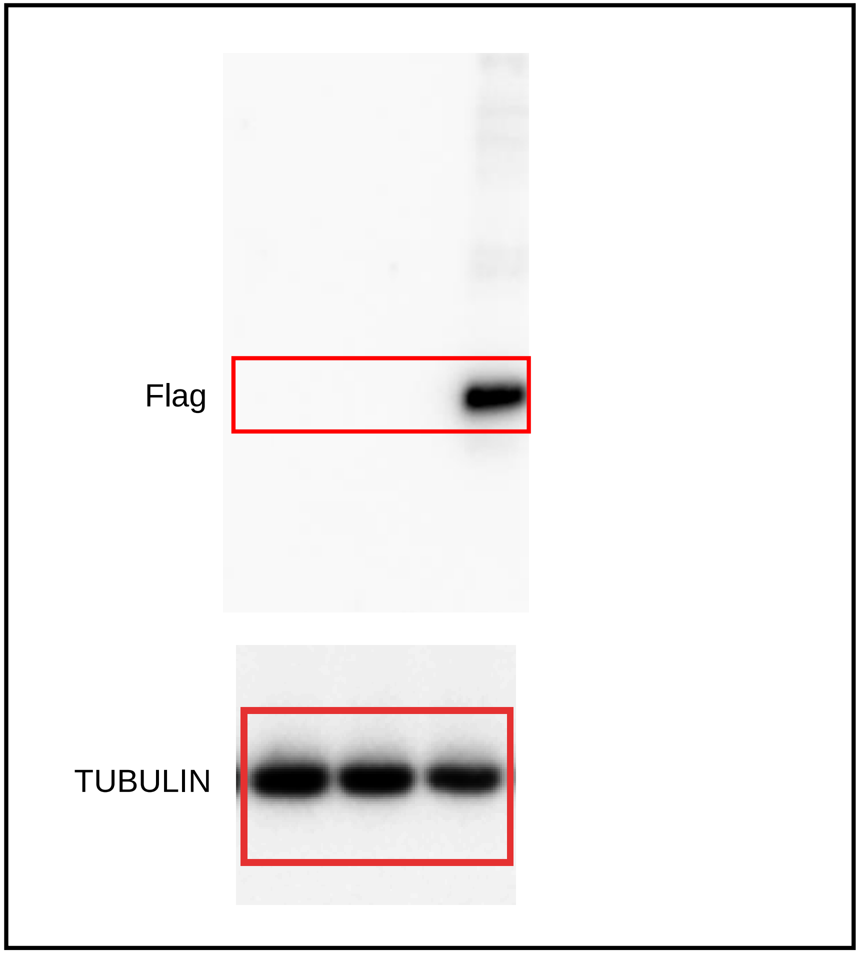

Fig. 3E

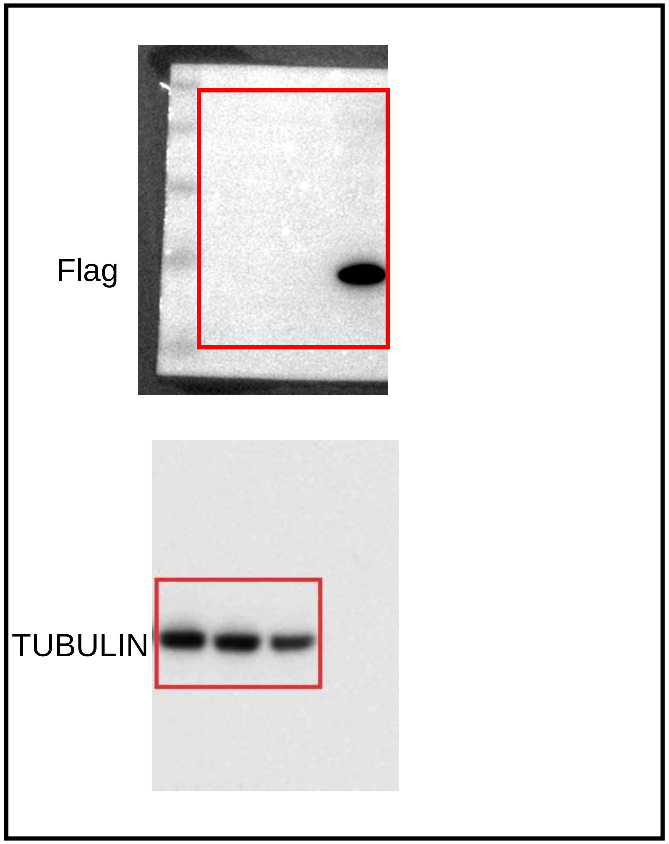

Fig. 4E

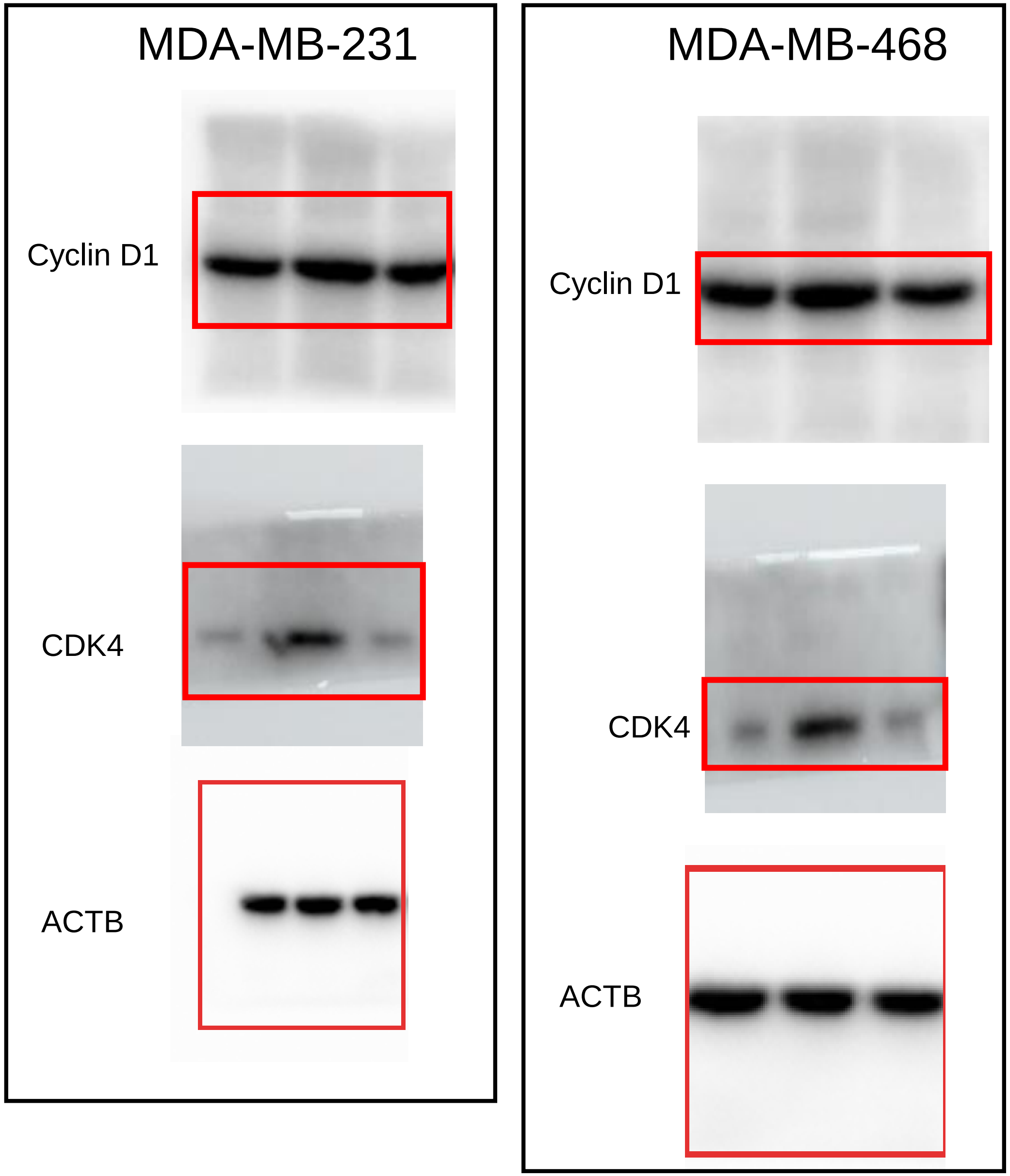

Fig. 5B

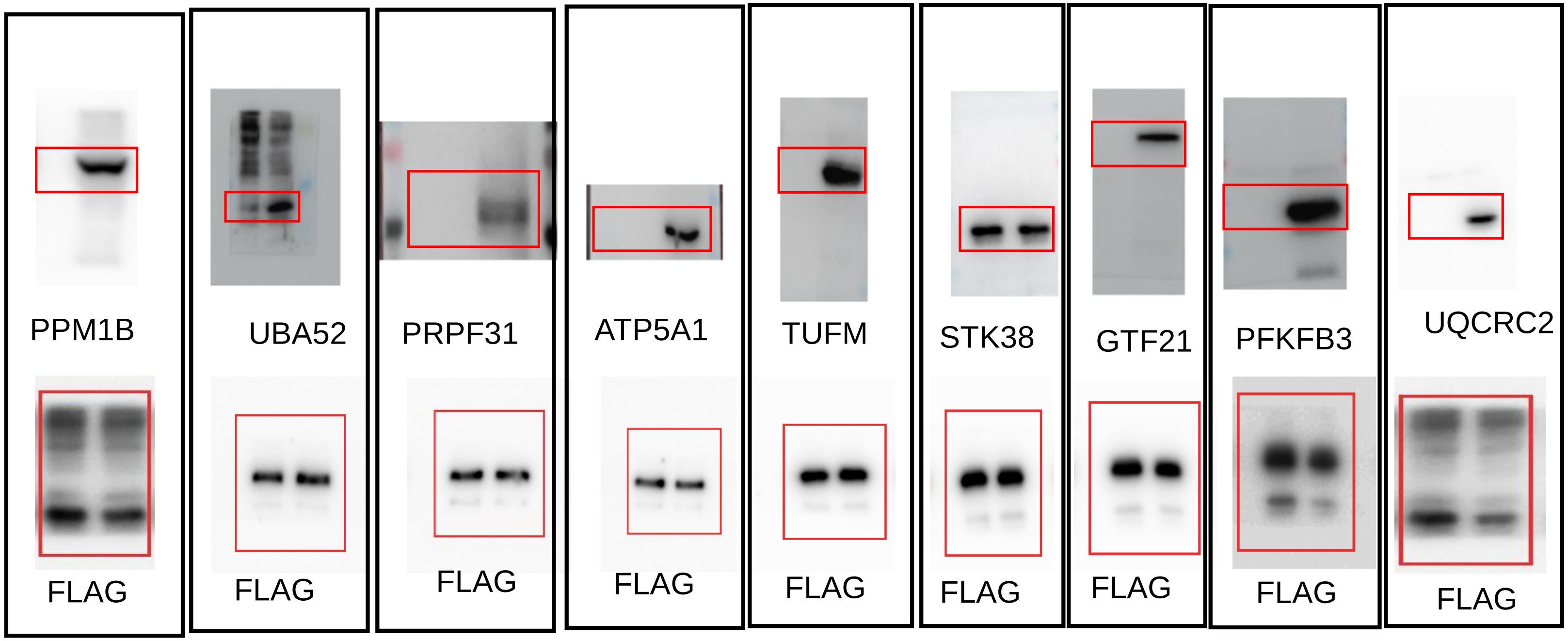
PPM1B
UBA52
PRPF31
ATP5A1
TUFM
STK38
GTF21
PFKFB3
UQCRC2
FLAG
FLAG
FLAG
FLAG
FLAG
FLAG
FLAG
FLAG
FLAG

Fig. 5C

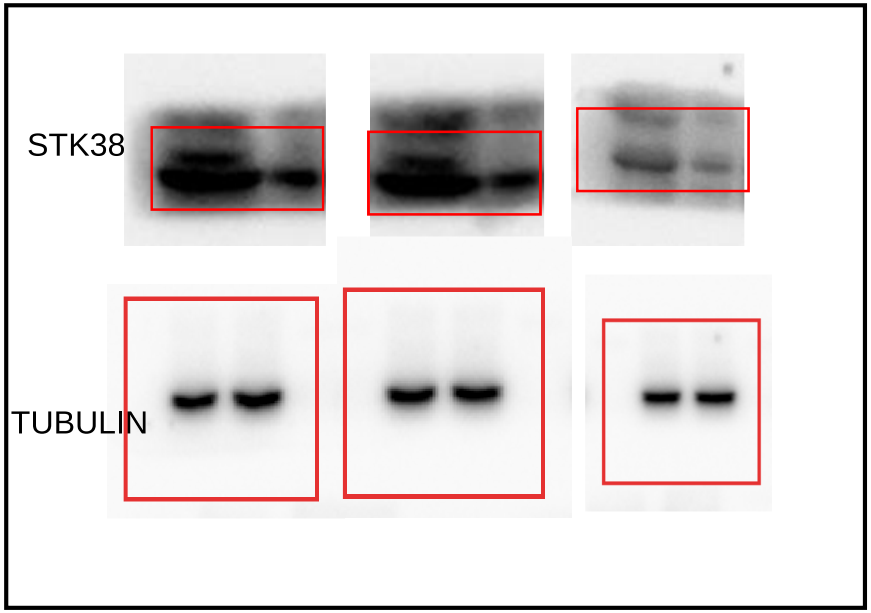

Fig. 5G

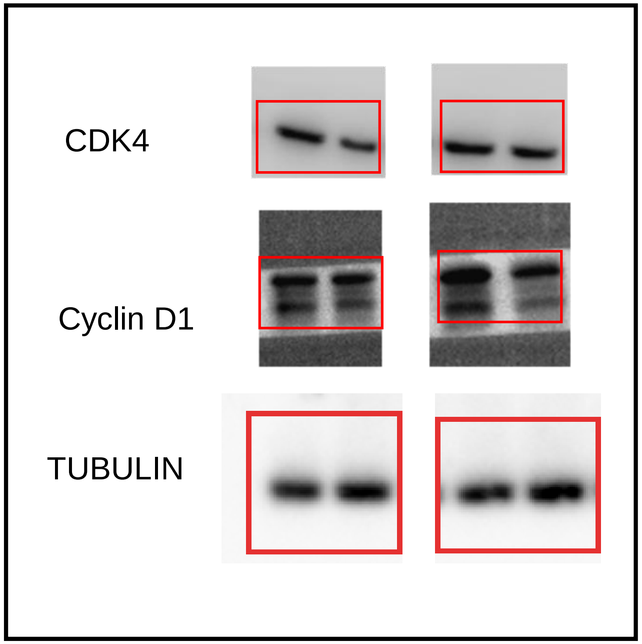

Fig. 6A

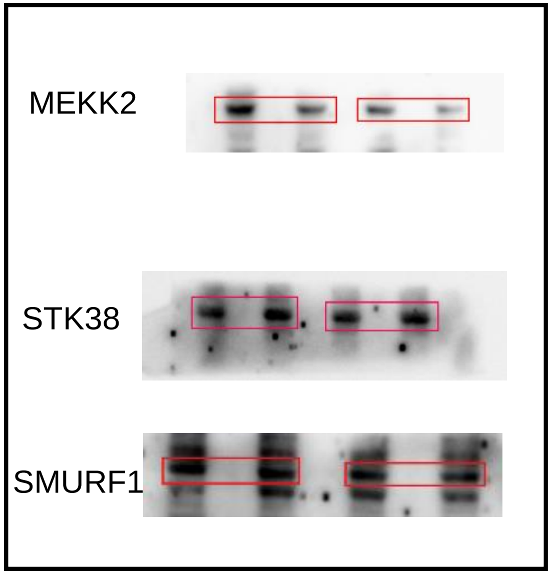

Fig. 6B

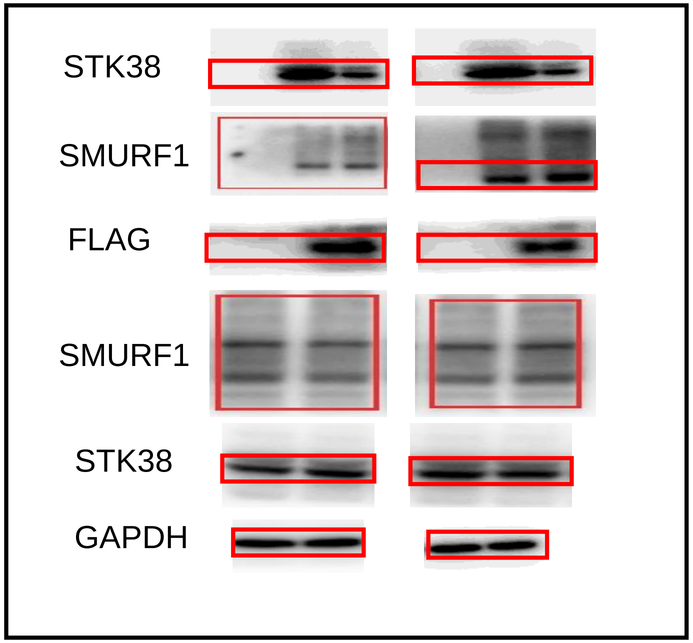

Fig. 6C

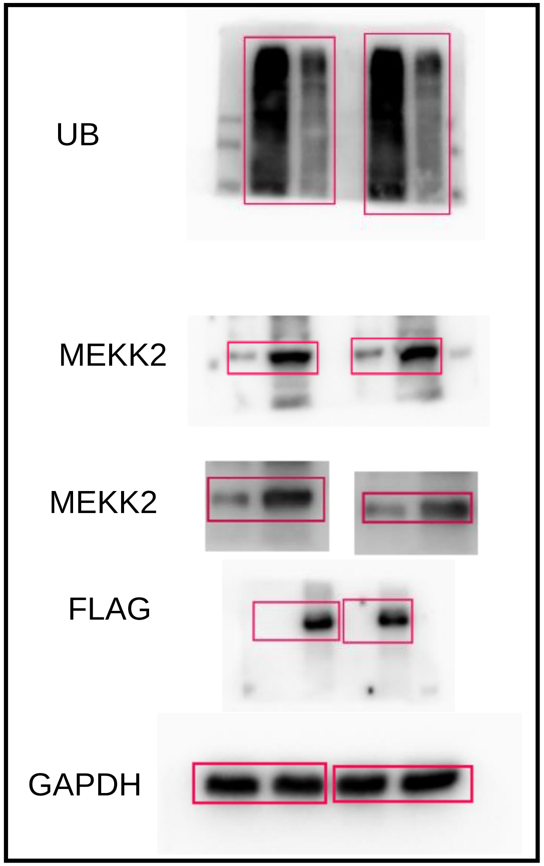

Fig. 6D

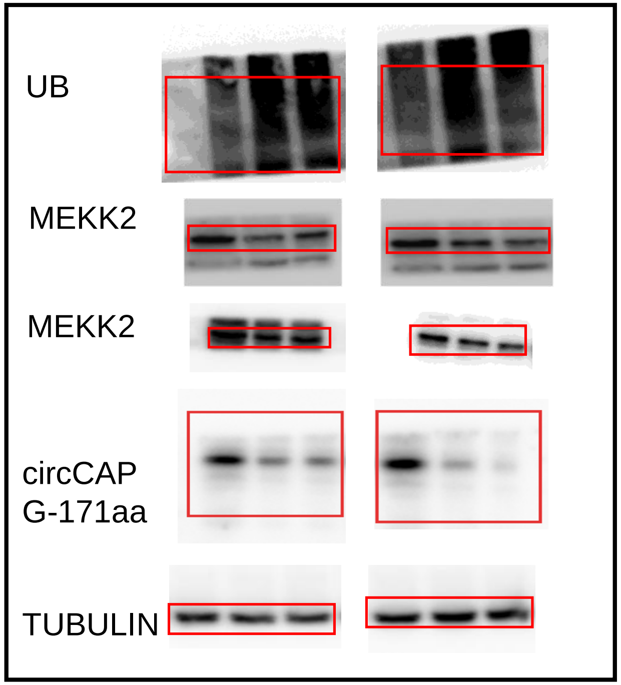

Fig. 6E

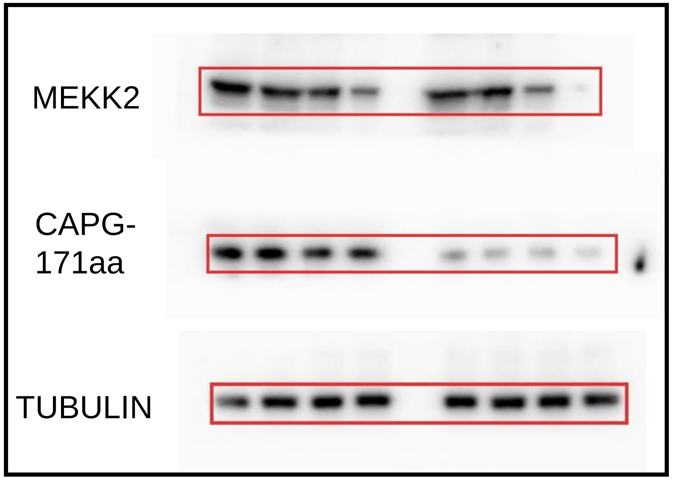

Fig. 6F

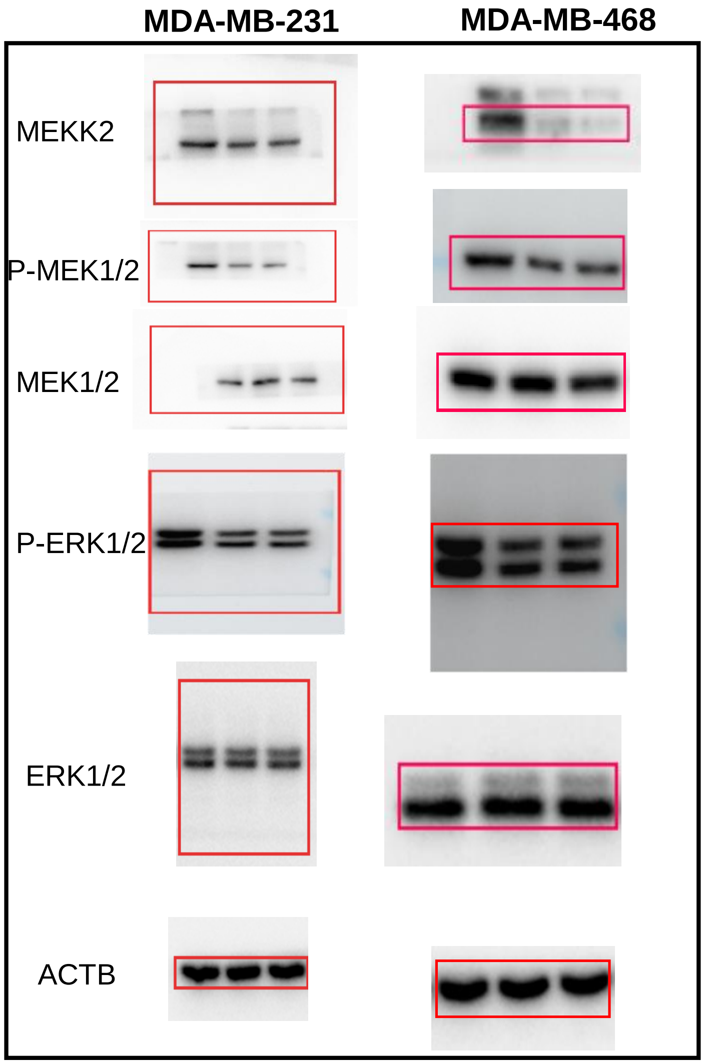

Fig. 6G

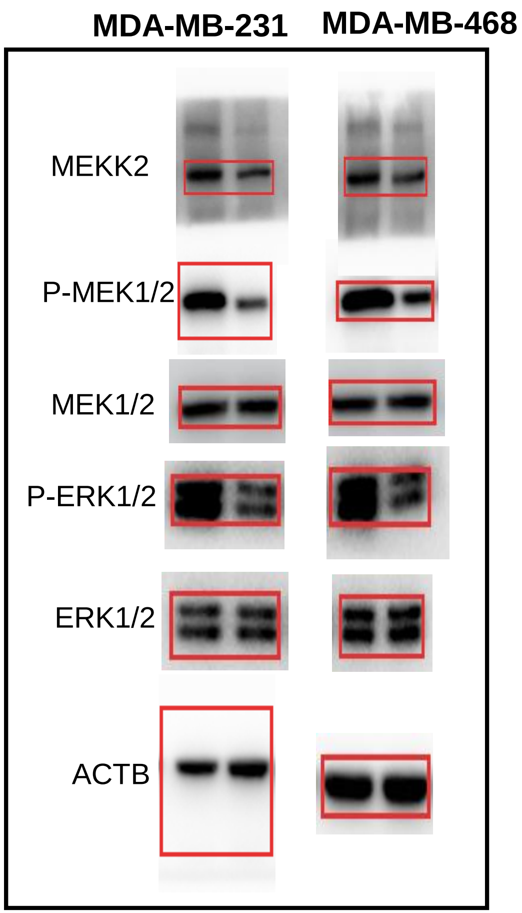

Fig. 6H

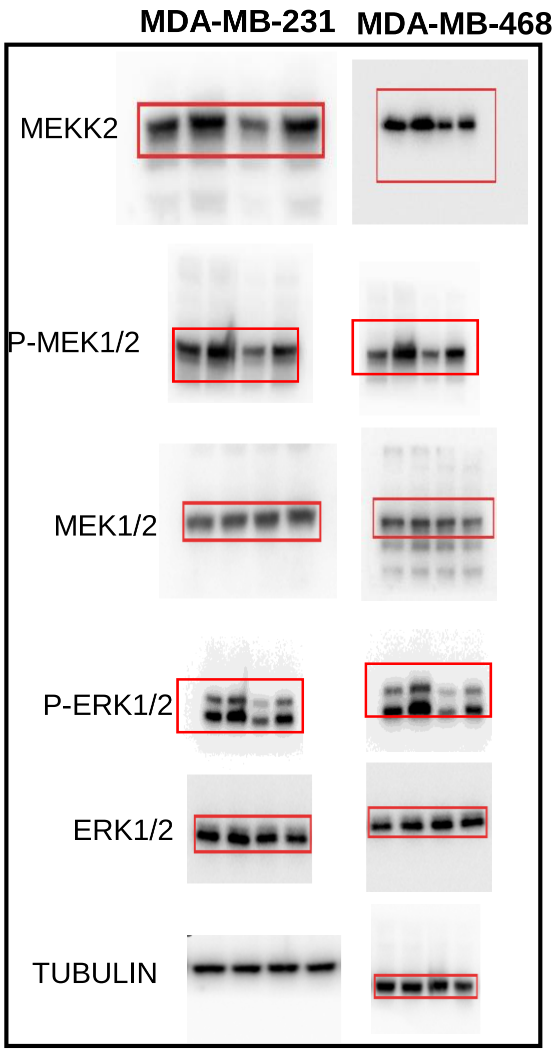

Fig. 7D

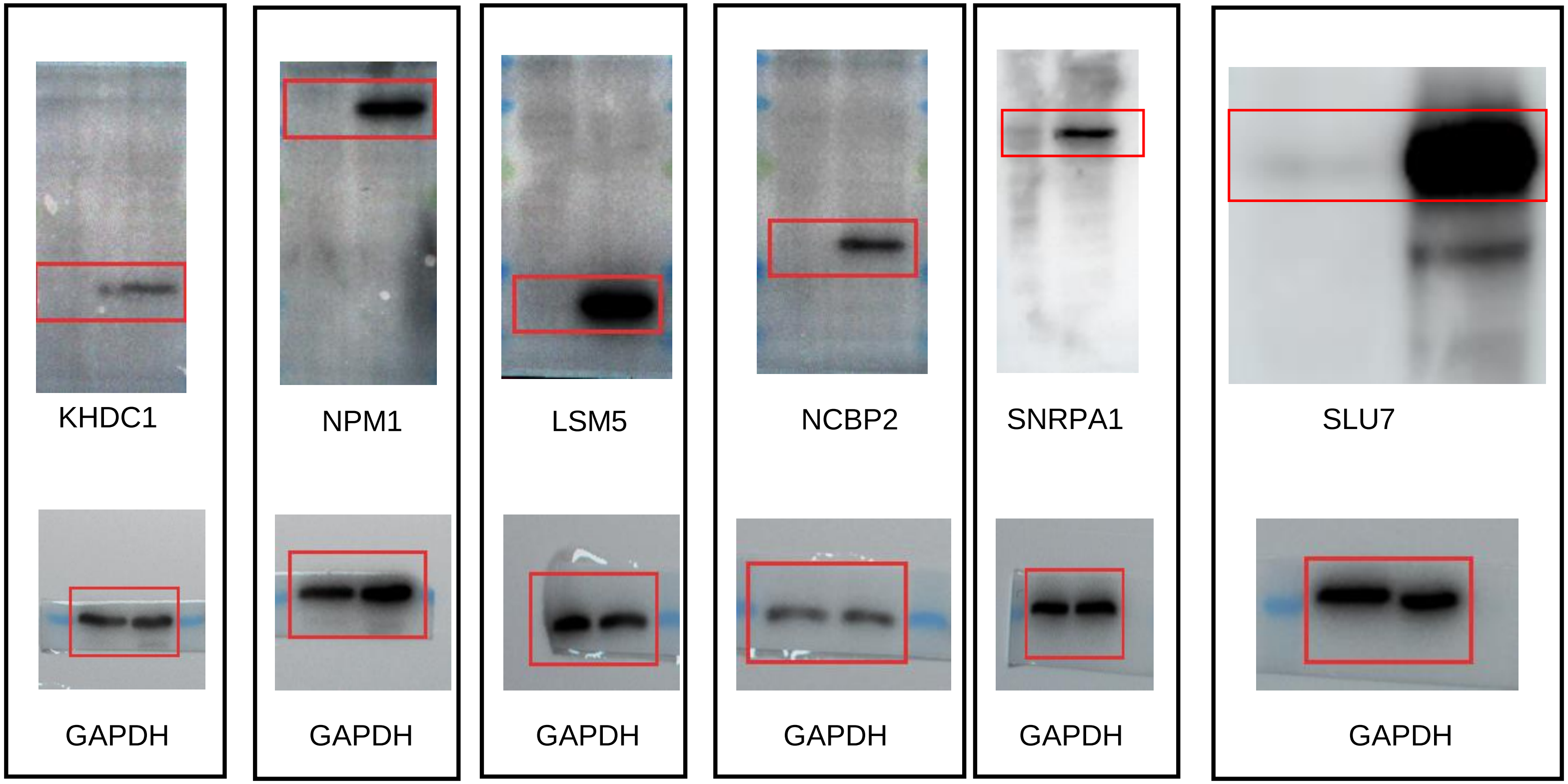
KHDC1
NPM1
LSM5
NCBP2
SNRPA1
SLU7
GAPDH
GAPDH
GAPDH
GAPDH
GAPDH
GAPDH

Fig. 8A

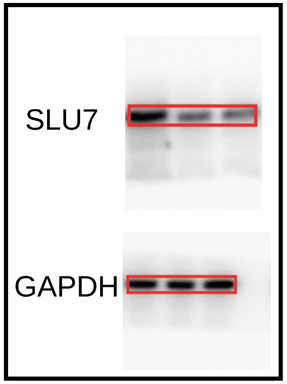

Fig. 8B

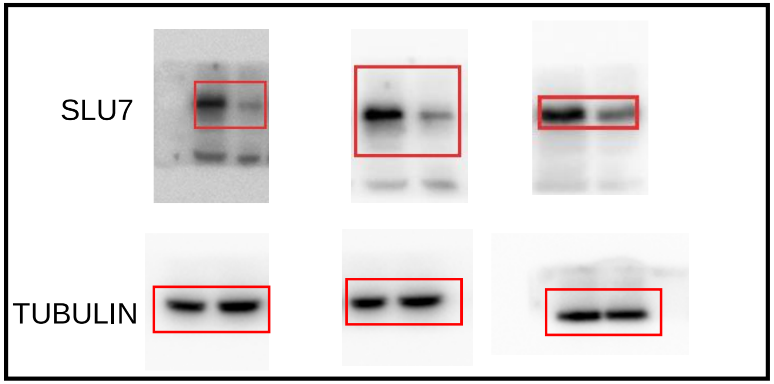

Fig. 8G

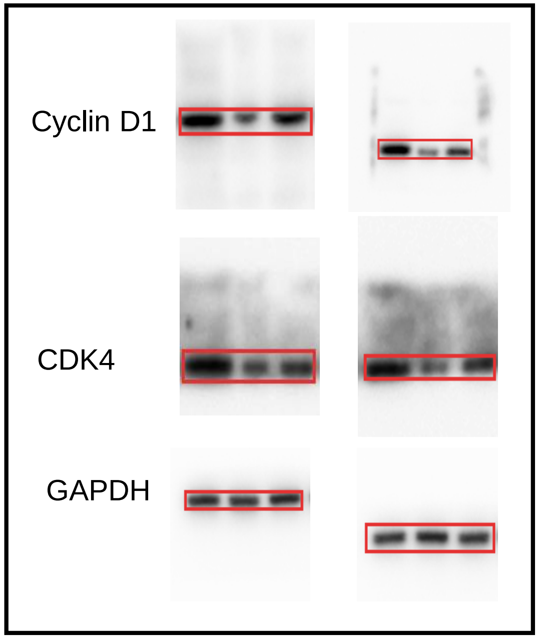

Fig. S2B

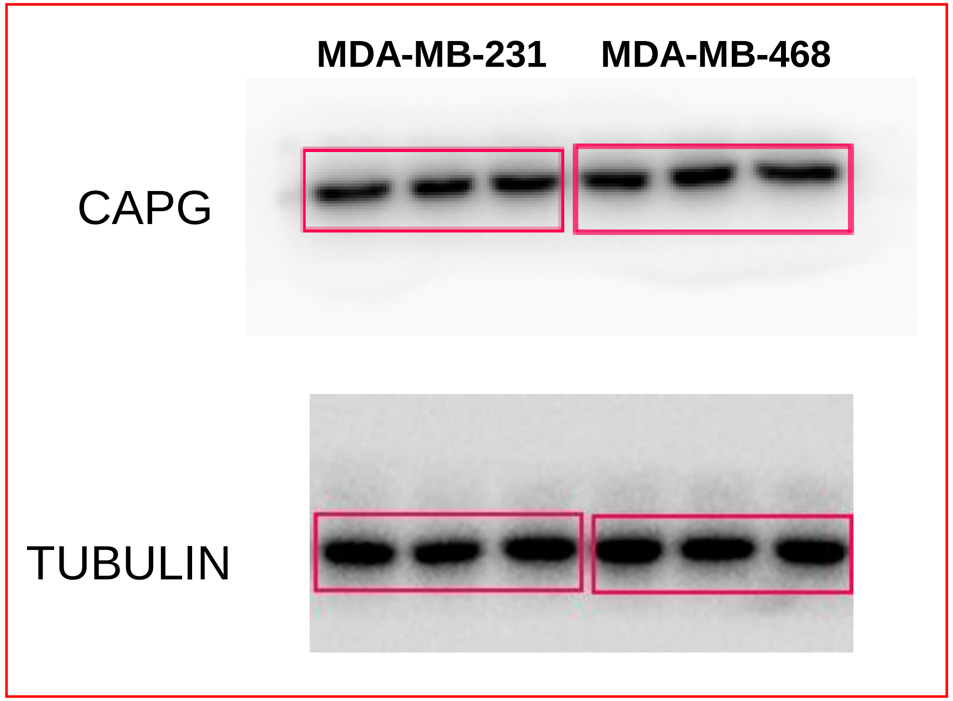

Fig. S2C

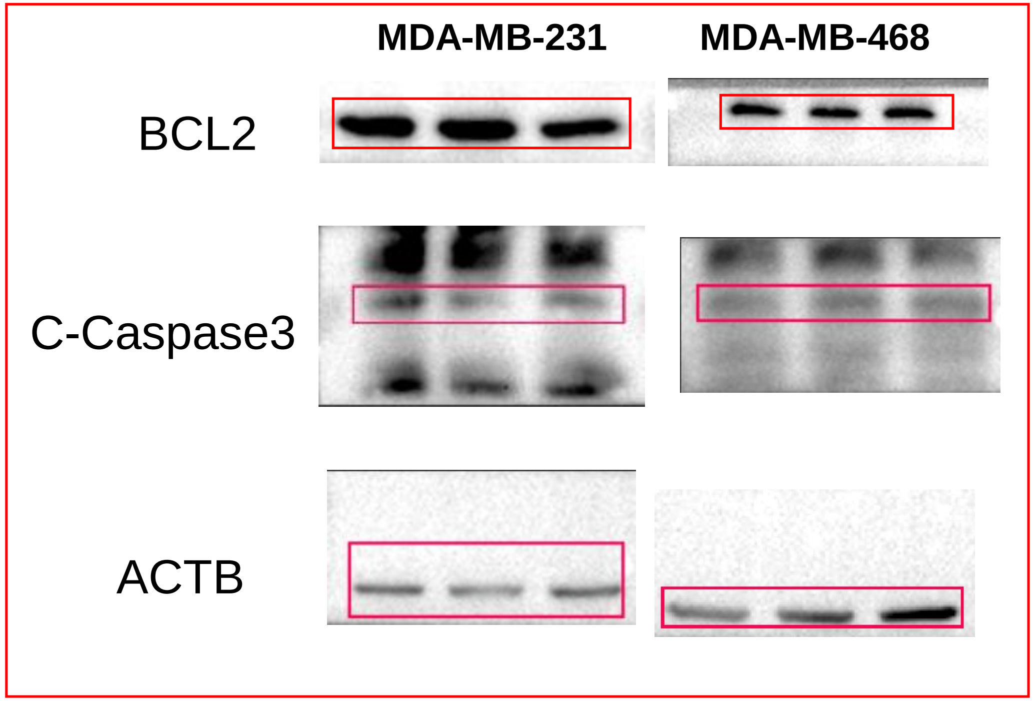

Fig. S3C

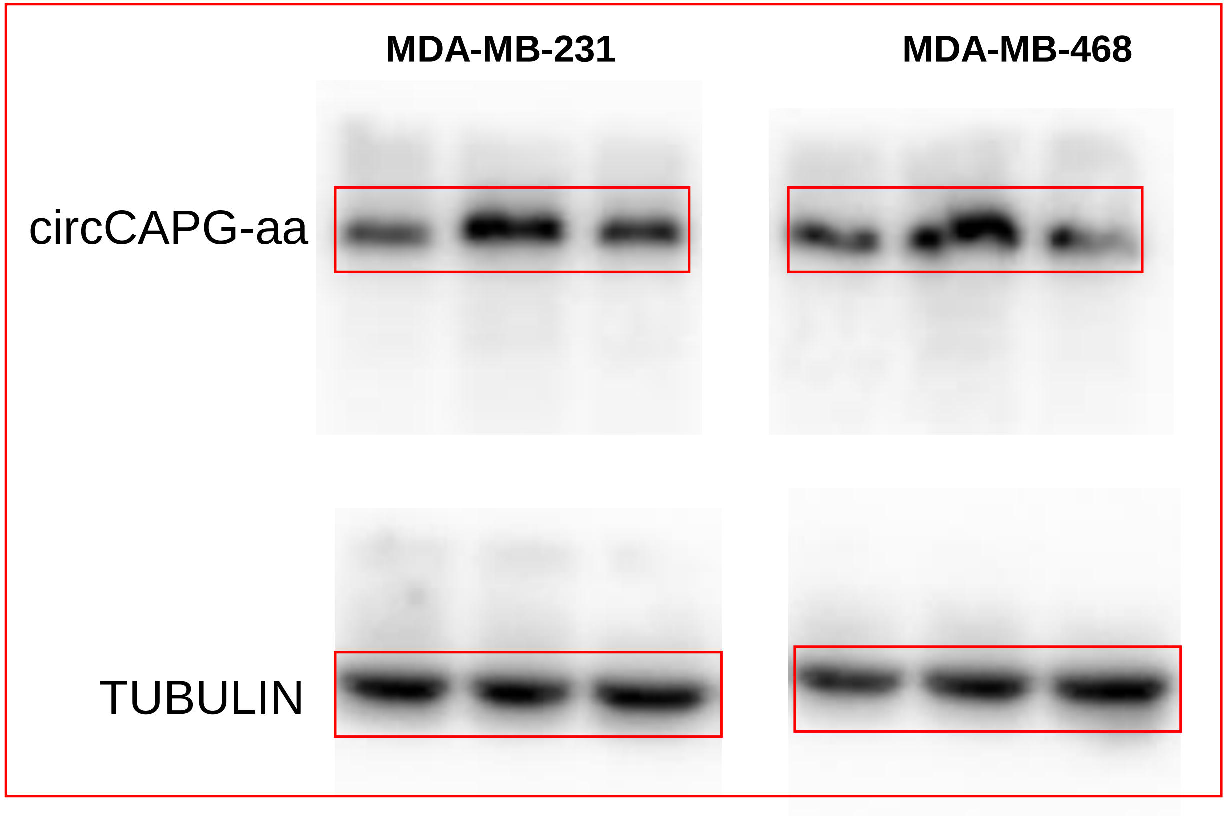

Fig. S4A

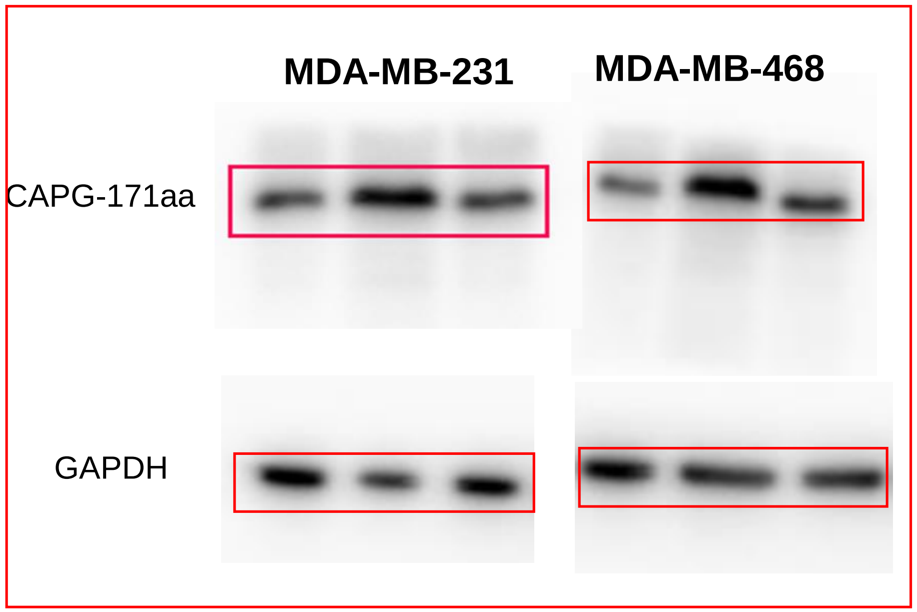

Fig. S4B

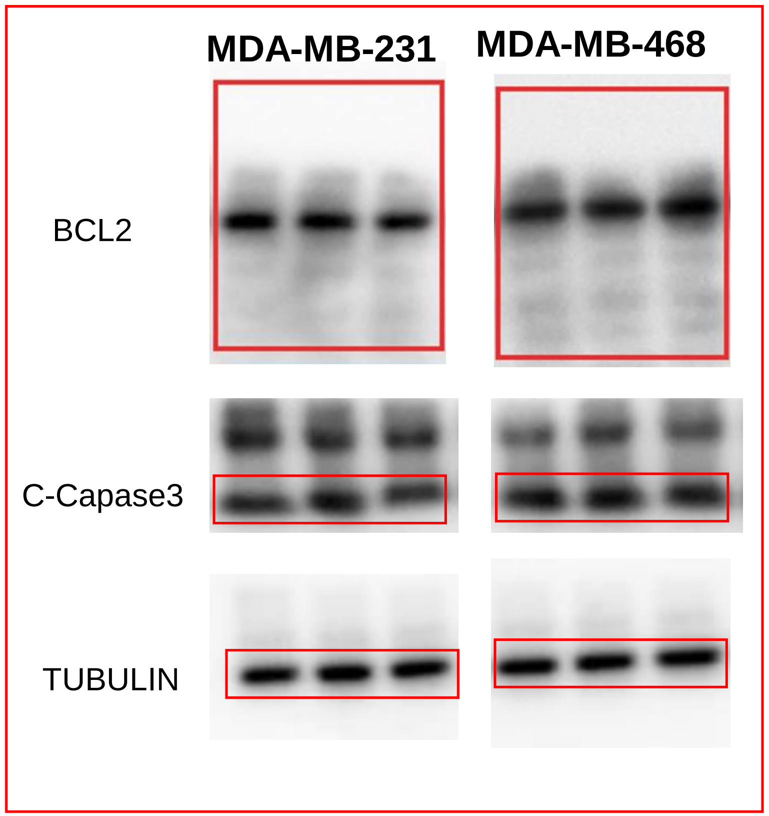

Fig. S5B

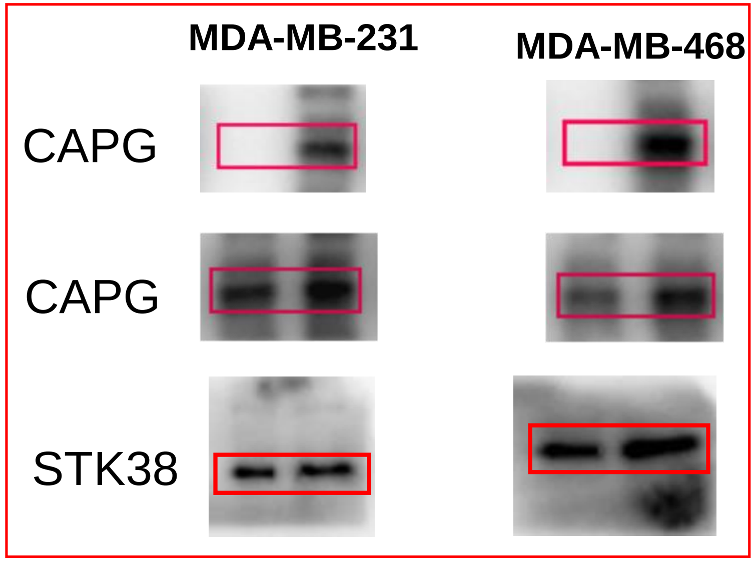

Fig. S5C

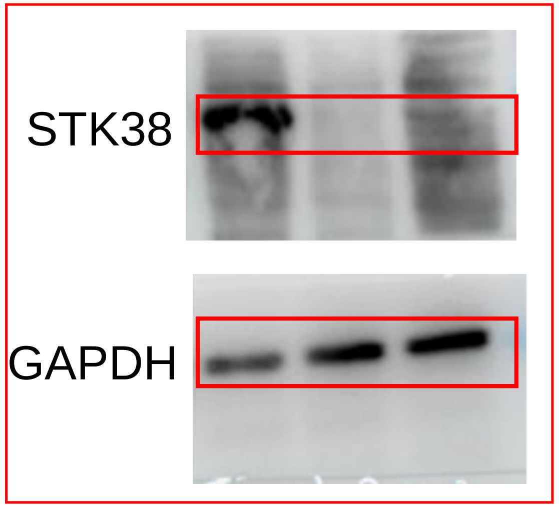

Fig. S5D

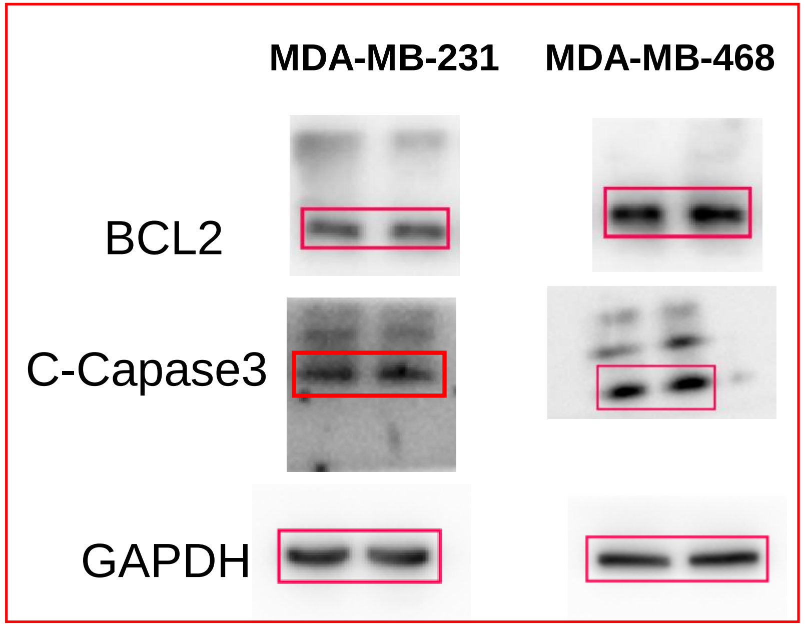

Fig. S5E

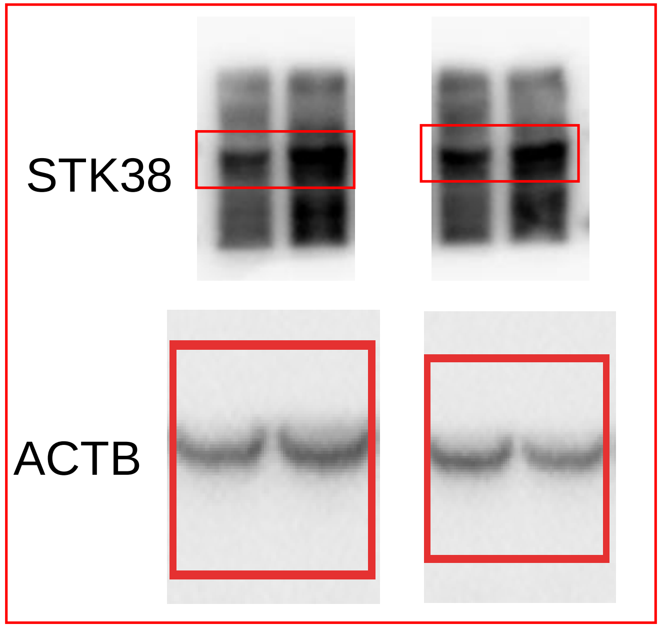

Fig. S6A

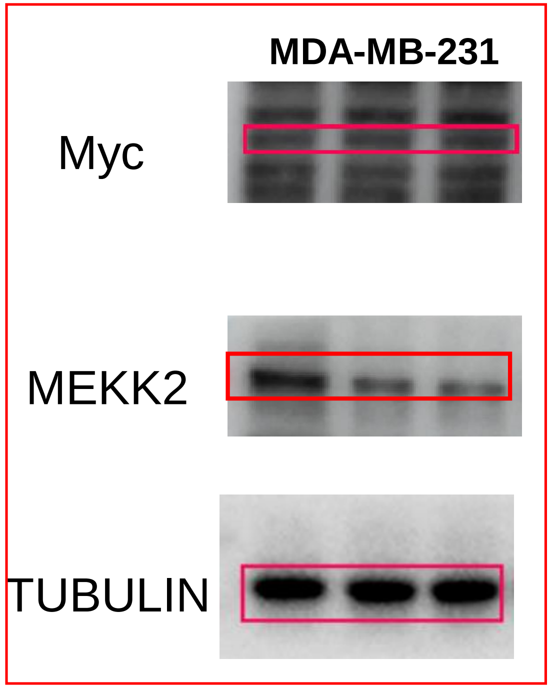

Fig. S6D

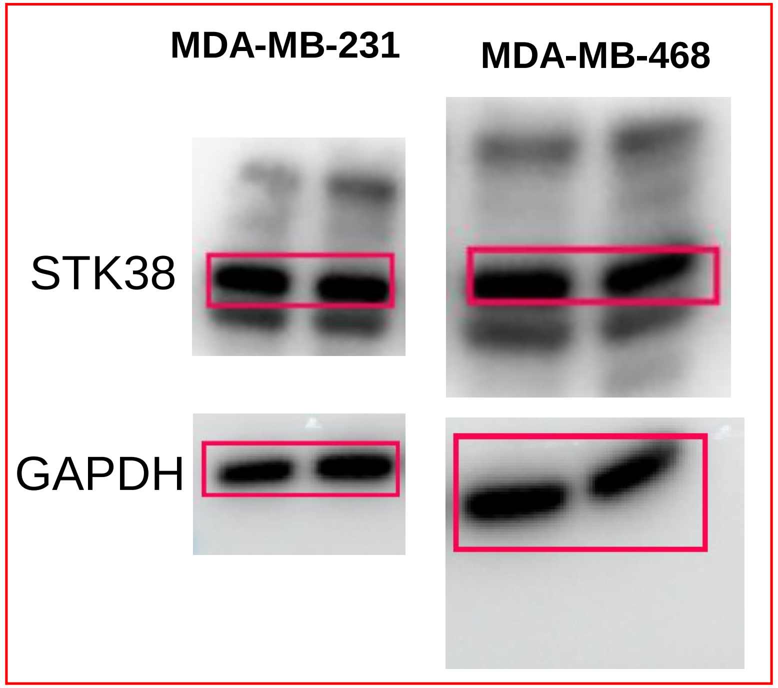

Fig. S6F

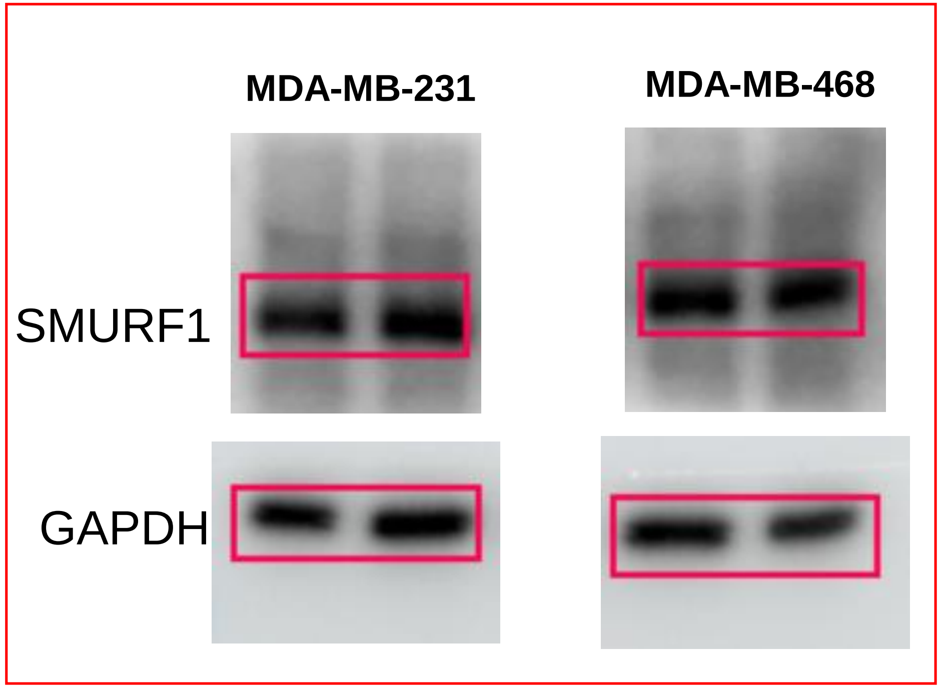

Fig. S6G

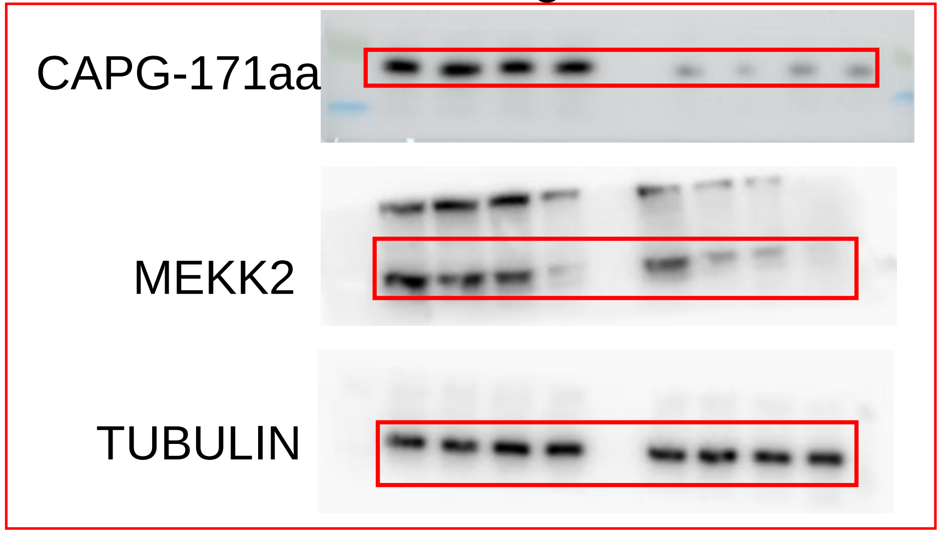

Fig. S7C

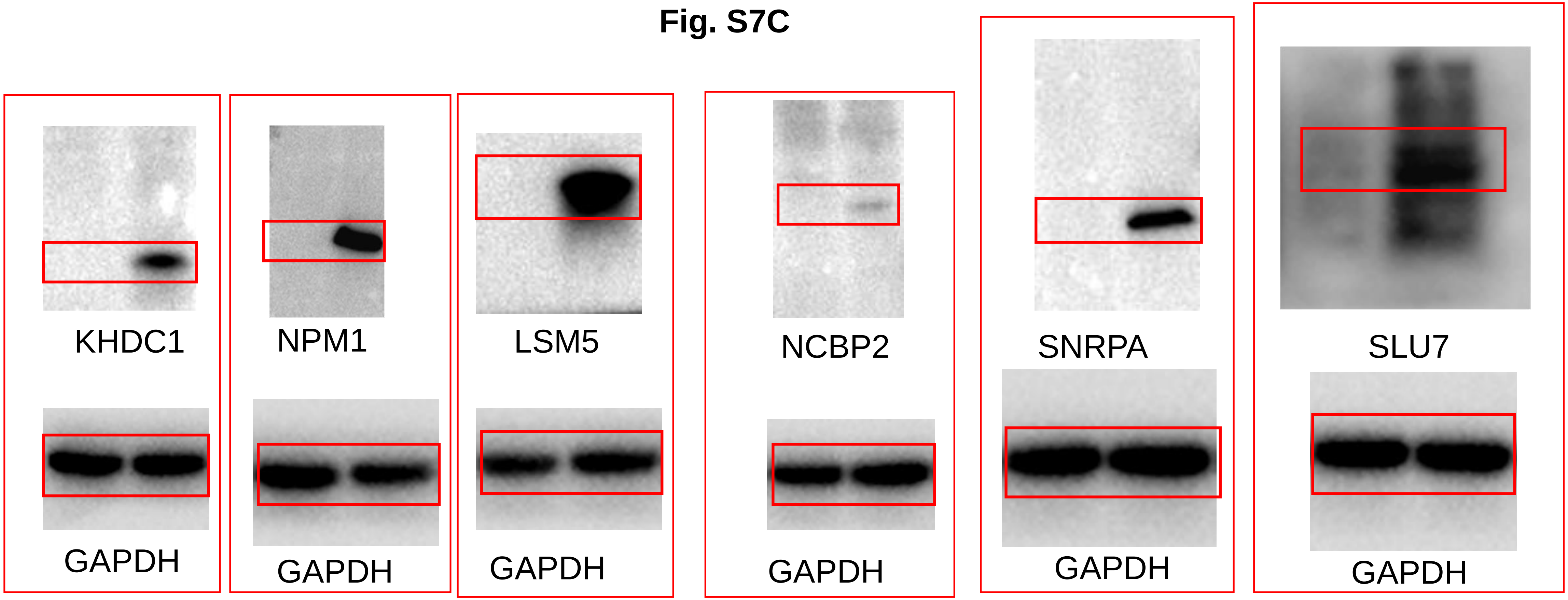

KHDC1
GAPDH
NPM1
GAPDH
LSM5
GAPDH
NCBP2
GAPDH
SNRPA
GAPDH
SLU7
GAPDH

Fig. S7I

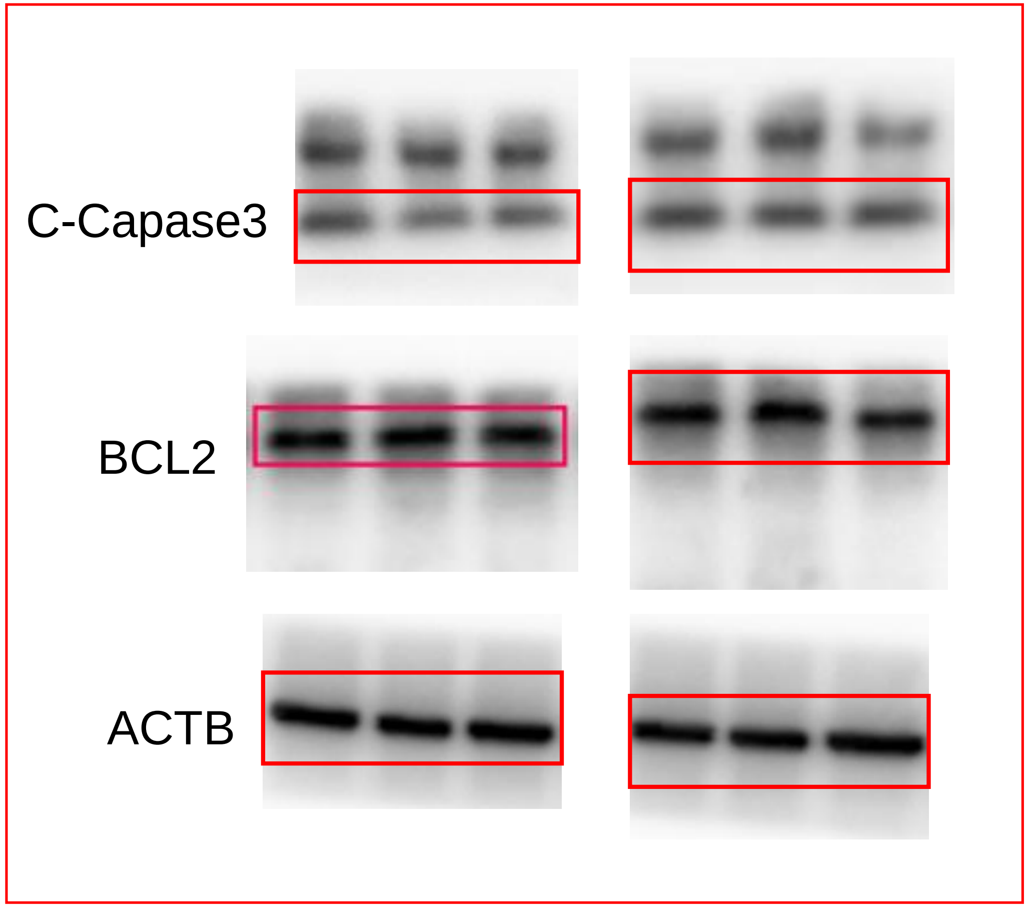

Supplement: Supplementary file 2 — Supplementary Material 2 [file 12943_2023_1806_MOESM2_ESM.pdf]
