# Supplementary material for: A novel polypeptide CAPG-171aa encoded by circCAPG plays a critical role in triple-negative breast cancer
Source: Mol Cancer. 2023 Jul 5;22:104. doi: 10.1186/s12943-023-01806-x (PMC10320902; doi:10.1186/s12943-023-01806-x)
Supplement: Supplementary file 3 — Supplementary Material 3 [file 12943_2023_1806_MOESM3_ESM.pdf]

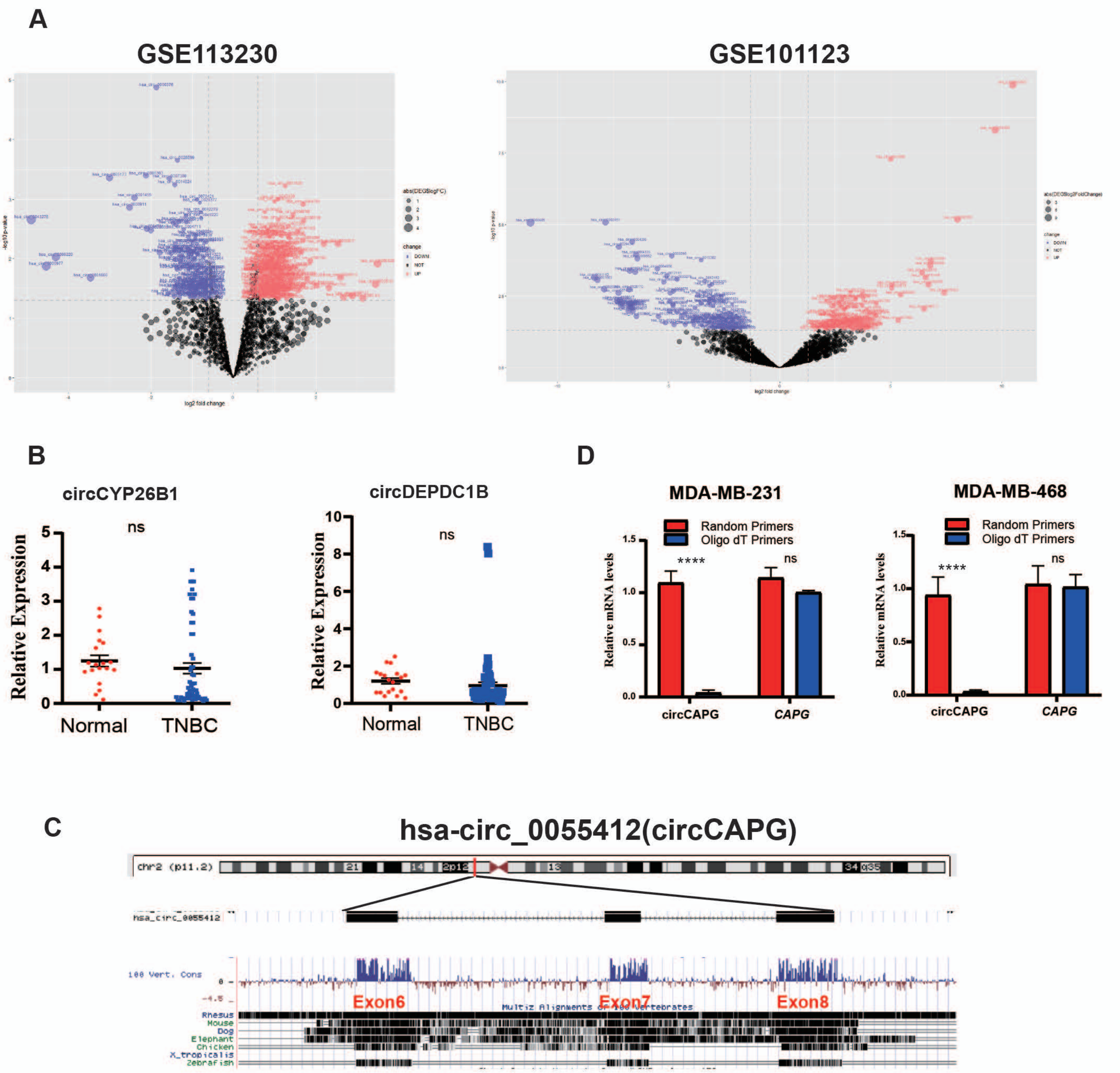
A
GSE113230
GSE101123
B
circCYP26B1
ns
Relative Expression
Normal
TNBC
circDEPDC1B
ns
Relative Expression
Normal
TNBC
D
MDA-MB-231
Random Primers
Oligo dT Primers
Relative mRNA levels
****
ns
circCAPG
CAPG
MDA-MB-468
Random Primers
Oligo dT Primers
Relative mRNA levels
****
ns
circCAPG
CAPG
C
hsa-circ_0055412(circCAPG)
Exon6
Exon7
Exon8

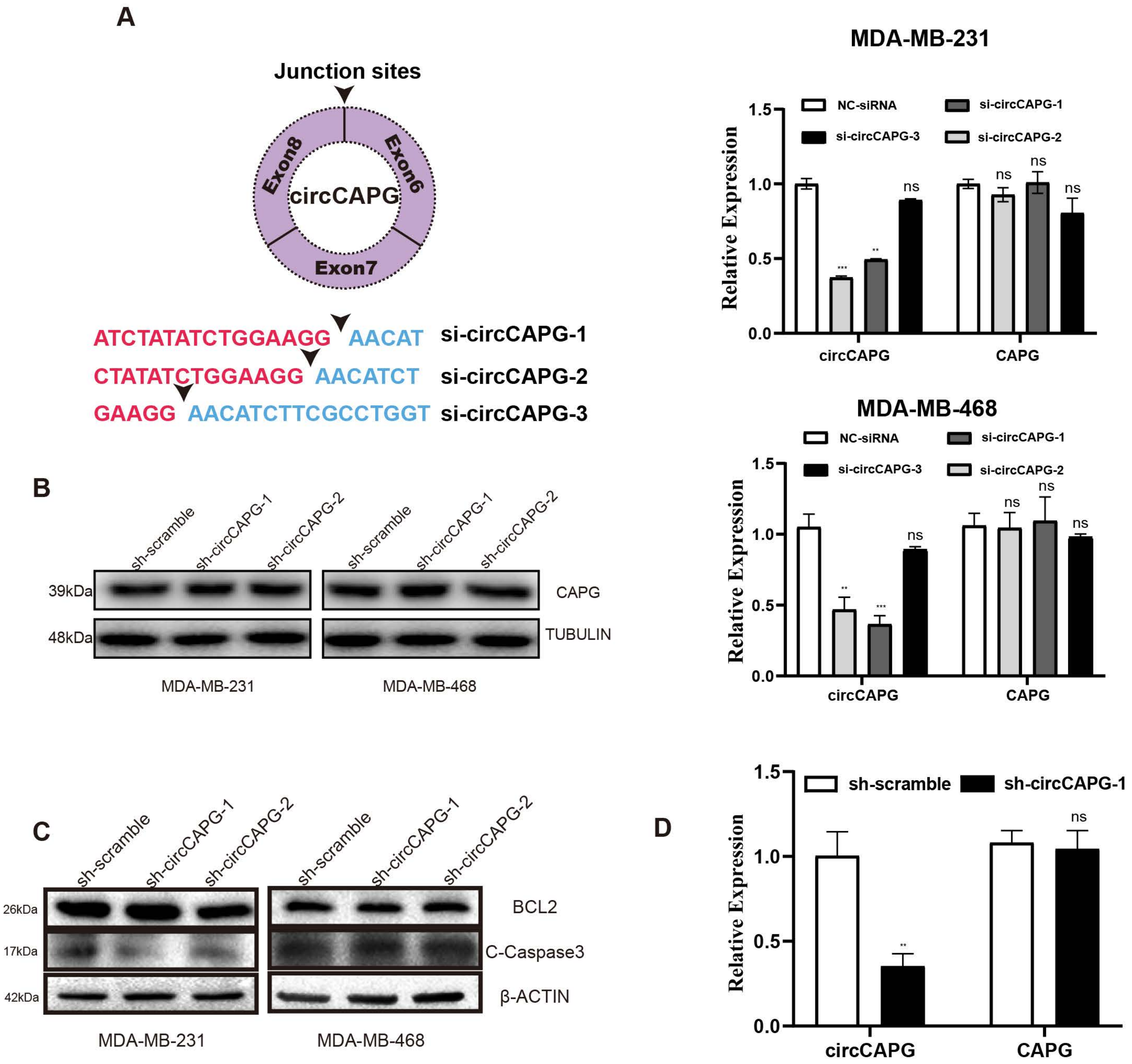
A
Junction sites
circCAPG
Exon8
Exon6
Exon7
ATCTATATCTGGAAGG AACAT si-circCAPG-1
CTATATCTGGAAGG AACATCT si-circCAPG-2
GAAGG AACATCTTCGCCTGGT si-circCAPG-3
MDA-MB-231
NC-siRNA
si-circCAPG-1
si-circCAPG-3
si-circCAPG-2
Relative Expression
circCAPG
CAPG
ns
MDA-MB-468
B
sh-scramble
sh-circCAPG-1
sh-circCAPG-2
39kDa
48kDa
CAPG
TUBULIN
MDA-MB-231
MDA-MB-468
C
26kDa
17kDa
42kDa
BCL2
C-Caspase3
β-ACTIN
D
sh-scramble
sh-circCAPG-1

A

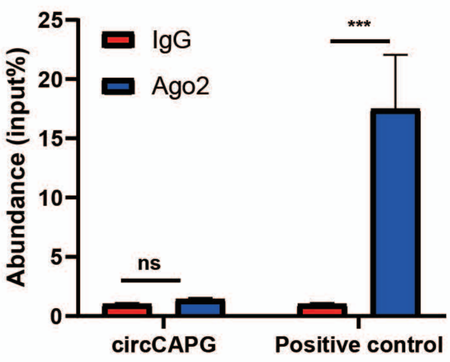

B

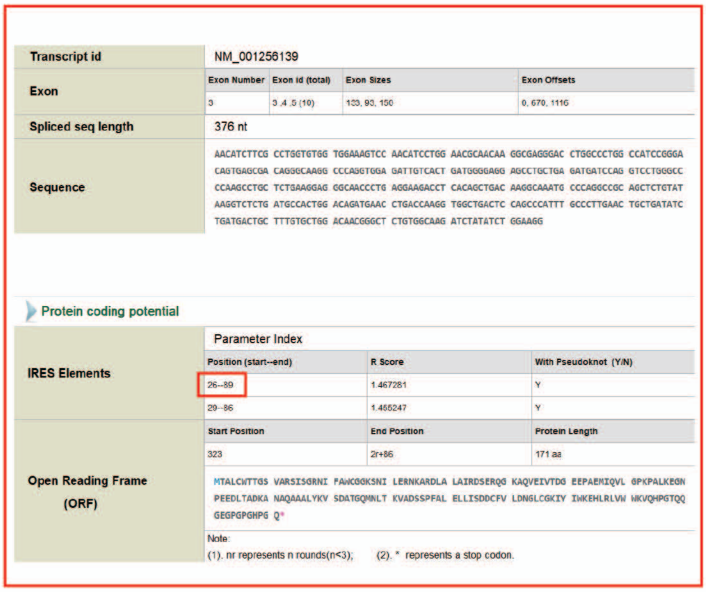

Transcript id	NM_001256139			
Exon	Exon Number	Exon id (total)	Exon Sizes	Exon Offsets
	3	3 ,4 ,5 (10)	133, 93, 150	0, 670, 1116
Spliced seq length	376 nt			
Sequence	AACATCTTCG CCTGGTGTGG TGGAAAGTCC AACATCCTGG AACGCAACAA GGCGAGGGAC CTGGCCCTGG CCATCCGGGA CAGTGAGCGA CAGGGCAAGG CCCAGGTGGA GATTGTCACT GATGGGGAGG AGCCTGCTGA GATGATCCAG GTCCTGGGCC CCAAGCCTGC TCTGAAGGAG GGCAACCCTG AGGAAGACCT CACAGCTGAC AAGGCAAATG CCCAGGCCGC AGCTCTGTAT AAGGTCTCTG ATGCCACTGG ACAGATGAAC CTGACCAAGG TGGCTGACTC CAGCCCATTT GCCCTTGAAC TGCTGATATC TGATGACTGC TTTGTGCTGG ACAACGGGCT CTGTGGCAAG ATCTATATCT GGAAGG			

Protein coding potential

IRES Elements	Parameter Index		
	Position (start--end)	R Score	With Pseudoknot (Y/N)
	26--89	1.467281	Y
	29--86	1.455247	Y
Open Reading Frame (ORF)	Start Position	End Position	Protein Length
	323	2r+86	171 aa
	MTALCWTTGS VARSISGRNI FAWCGGKSNI LERNKARDLA LAIRDSERQG KAQVEIVTDG EEPAEMIQVL GPKPALKEGN PEEDLTADKA NAQAAALYKV SDATGQMNLT KVADSSPFAL ELLISDDCFV LDNGLCGKIY IWKEHLRLVW WKVQHPGTQQ GEGPGPGHPG Q*		
	Note: (1). nr represents n rounds(n<3); (2). * represents a stop codon.		

C

CAPG full length
MYTAIPQSGSPFPGSVQDPGLHVWRVEKLKPVPVAQENQGVFFSGDSYLVLHNGPEEVSH
LHLWIGQQSSRDEQGACAVLAVHLNTLLGERPVQHREVQGNESDLFMSYFPRGLKYQEGG
VESAFHKTSTGAPAAIKKLYQVKGKKNIRATERALNWDSFNTGDCFILDLGQNIFAWCGG
KSNILERNKARDLALAIRDSERQGKAQVEIVTDGEEPAEMIQVLGPKPALKEGNPEEDLT
ADKANAQAAALYKVSDATGQMNLTKVADSSPFALELLISDDCFVLDNGLCGKIYIWKGRK
ANEKERQAALQVAEGFISRMQYAPNTQVEILPQGHESPIFKQFFKDWK

CAPG-171aa
MTALCWTTGS VARSISGRNI FAWCGGKSNI LERNKARDLA LAIRDSERQG KAQVEIVTDG
EEPAEMIQVL GPKPALKEGN PEEDLTADKA NAQAAALYKV SDATGQMNLT KVADSSPFAL
ELLISDDCFV LDNGLCGKIY IWKEHLRLVW WKVQHPGTQQ GEGPGPGHPG Q

CAPG Polyclonal antibody(Proteintech #10194-1-AP)
HLWIGQQSSRDEQGACAVLAVHLNTLLGERPVQHREVQGNESDLFMSYFPRGLKYQEGGV
ESAFHKTSTGAPAAIKKLYQVKGKKNIRATERALNWDSFNTGDCFILDLGQNIFAWCGGKSNI
LERNKARDLALAIRDSERQGKAQVEIVTDGEEPAEMIQVLGPKPALKEGNPEEDLTADKANA
QAAALYKVSDATGQMNLTKVADSSPFALELLISDDCFVLDNGLCGKIYIWKGRKAN
EKERQAALQVAEGFISRMQYAPNTQVEILPQGRESPIFKQFFKDWK
(62-348 aa encoded by BC000728)

Blue marked CAPG antibody targeting sequence

D

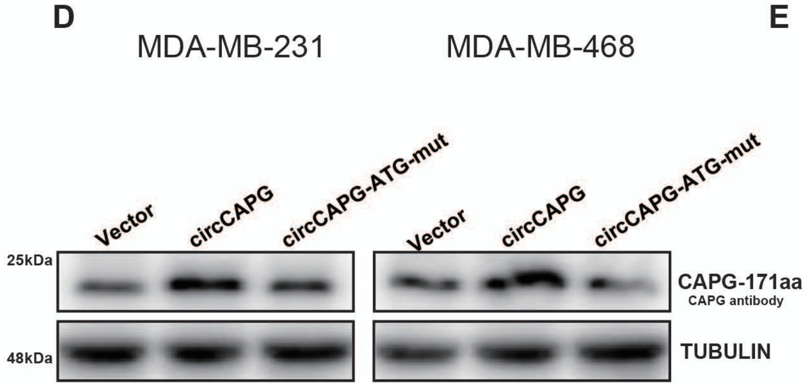

E

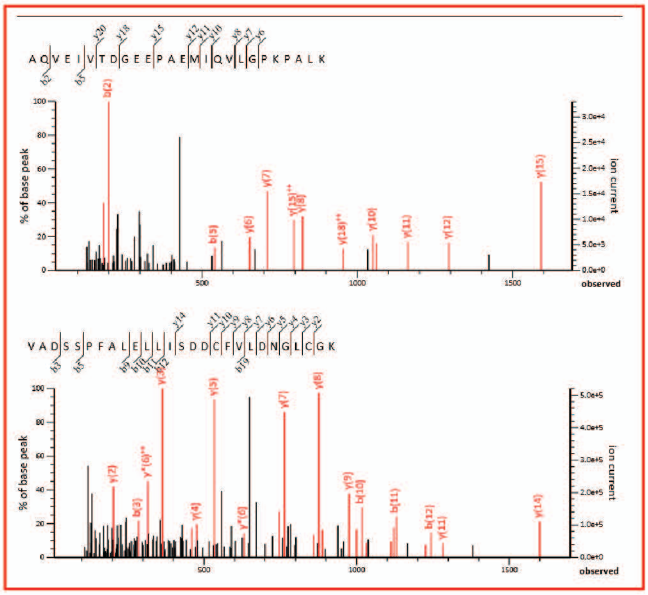

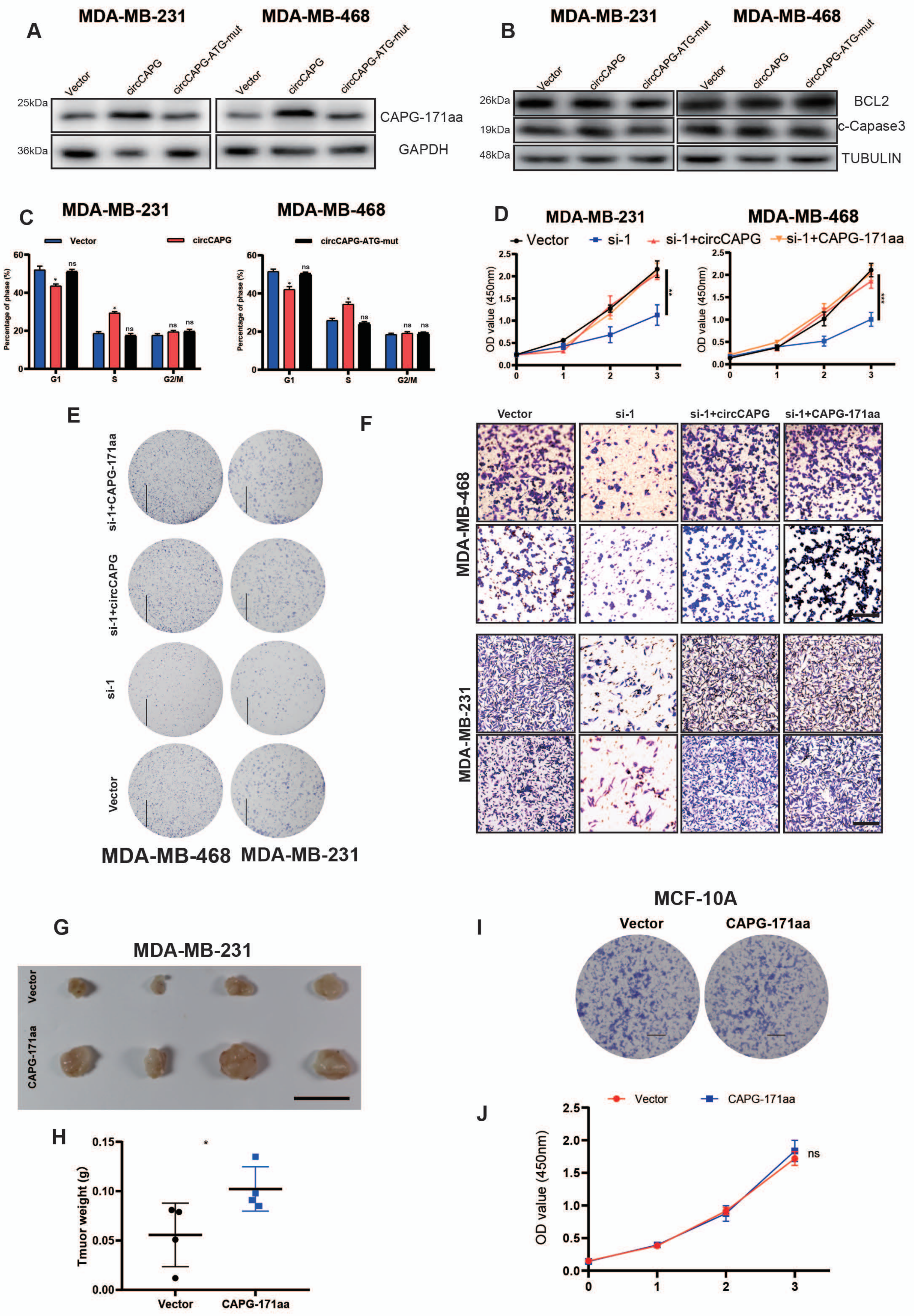

A
MDA-MB-231
MDA-MB-468
Vector
circCAPG
circCAPG-ATG-mut
25kDa
36kDa
CAPG-171aa
GAPDH
B
MDA-MB-231
MDA-MB-468
26kDa
19kDa
48kDa
BCL2
c-Capase3
TUBULIN
C
MDA-MB-231
MDA-MB-468
Vector
circCAPG
circCAPG-ATG-mut
Percentage of phase (%)
G1
S
G2/M
ns
D
MDA-MB-231
MDA-MB-468
Vector
si-1
si-1+circCAPG
si-1+CAPG-171aa
OD value (450nm)
E
si-1+CAPG-171aa
si-1+circCAPG
si-1
Vector
MDA-MB-468
MDA-MB-231
F
Vector
si-1
si-1+circCAPG
si-1+CAPG-171aa
MDA-MB-468
MDA-MB-231
G
MDA-MB-231
Vector
CAPG-171aa
H
Tmuor weight (g)
Vector
CAPG-171aa
I
MCF-10A
Vector
CAPG-171aa
J
Vector
CAPG-171aa
OD value (450nm)
ns

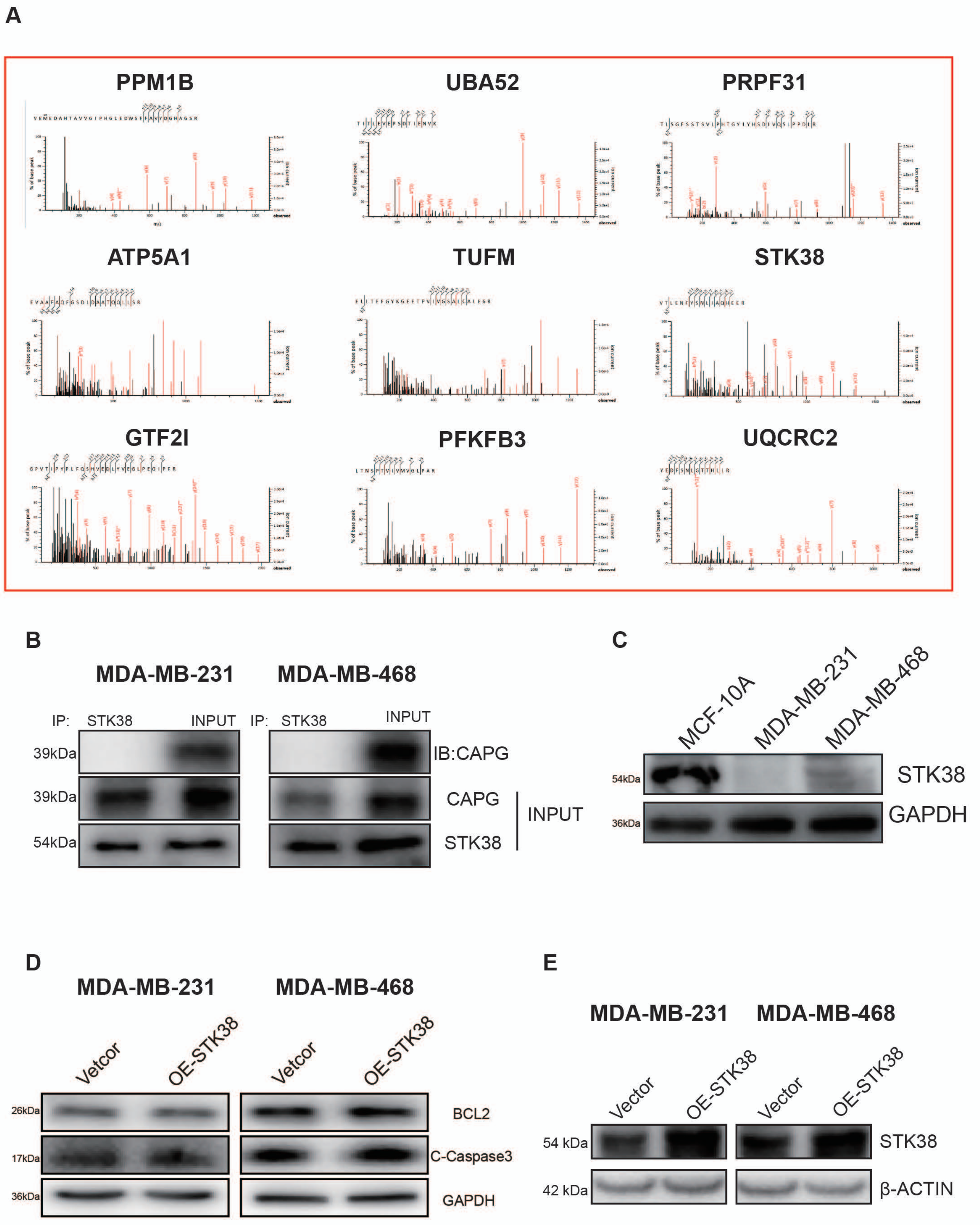
A
PPM1B
UBA52
PRPF31
ATP5A1
TUFM
STK38
GTF2I
PFKFB3
UQCRC2
B
MDA-MB-231
MDA-MB-468
IP: STK38
INPUT
IP: STK38
INPUT
39kDa
39kDa
54kDa
IB:CAPG
CAPG
STK38
INPUT
C
MCF-10A
MDA-MB-231
MDA-MB-468
54kDa
36kDa
STK38
GAPDH
D
MDA-MB-231
MDA-MB-468
Vetcor
OE-STK38
Vetcor
OE-STK38
26kDa
17kDa
36kDa
BCL2
C-Caspase3
GAPDH
E
MDA-MB-231
MDA-MB-468
Vector
OE-STK38
Vector
OE-STK38
54 kDa
42 kDa
STK38
β-ACTIN

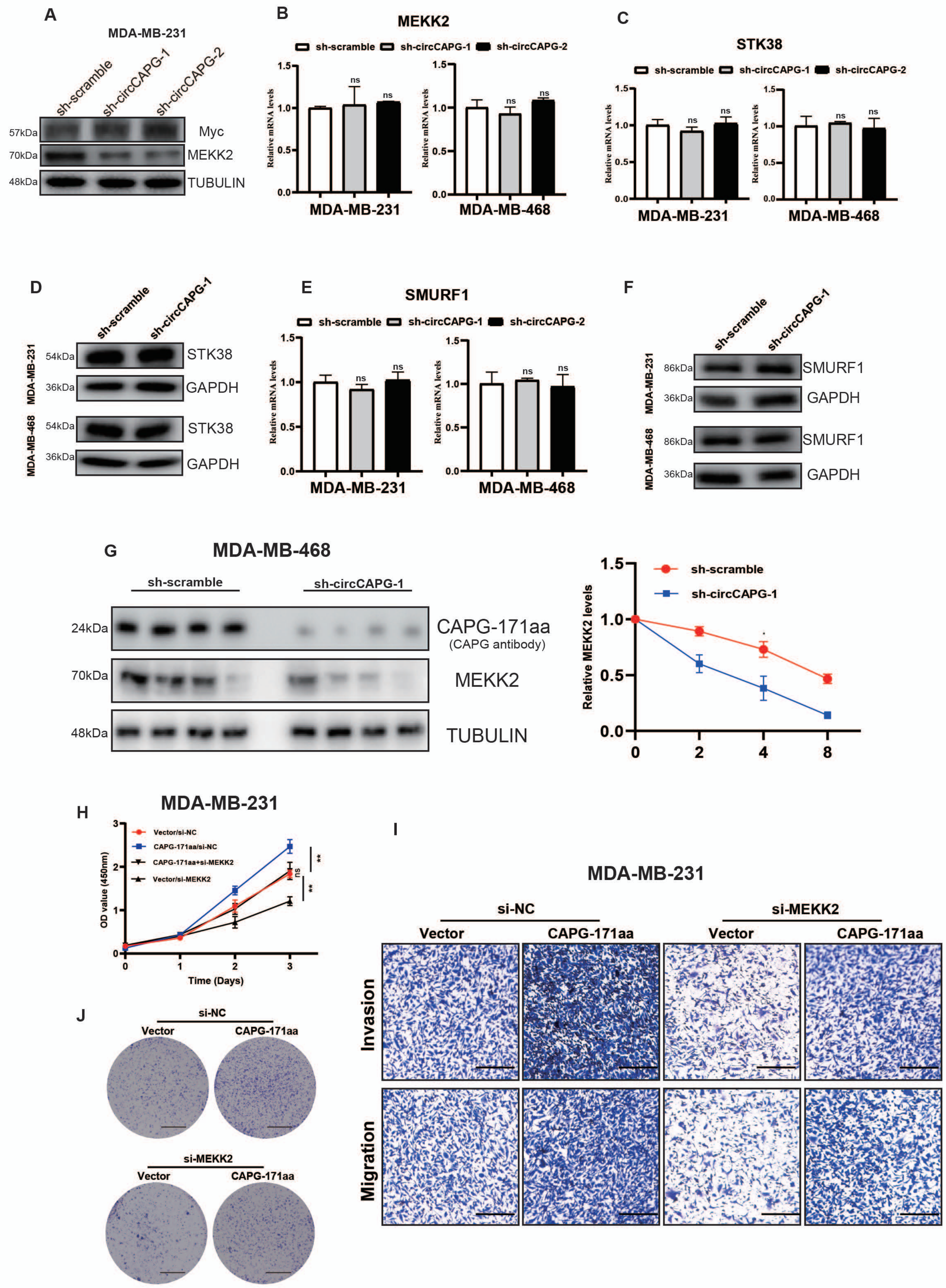
A
MDA-MB-231
sh-scramble
sh-circCAPG-1
sh-circCAPG-2
57kDa
Myc
70kDa
MEKK2
48kDa
TUBULIN
B
MEKK2
sh-scramble
sh-circCAPG-1
sh-circCAPG-2
Relative mRNA levels
ns
MDA-MB-231
MDA-MB-468
C
STK38
D
54kDa
STK38
36kDa
GAPDH
E
SMURF1
F
86kDa
SMURF1
G
MDA-MB-468
CAPG-171aa
(CAPG antibody)
24kDa
Relative MEKK2 levels
H
MDA-MB-231
Vector/si-NC
CAPG-171aa/si-NC
CAPG-171aa+si-MEKK2
Vector/si-MEKK2
OD value (450nm)
Time (Days)
**
I
si-NC
si-MEKK2
Vector
CAPG-171aa
Invasion
Migration
J

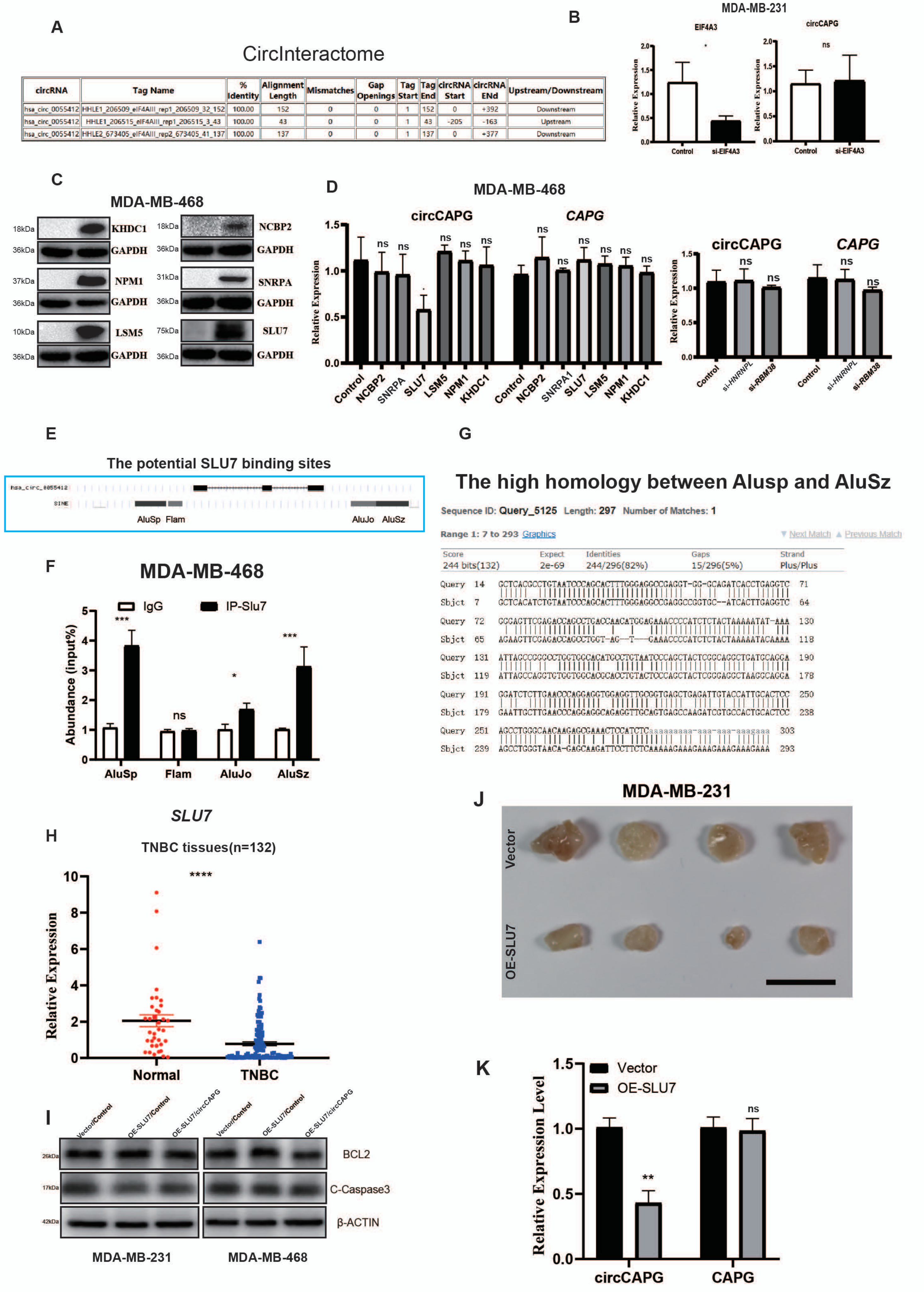

circRNA	Tag Name	% Identity	Alignment Length	Mismatches	Gap Openings	Tag Start	Tag End	circRNA Start	circRNA ENd	Upstream/Downstream
hsa_circ_0055412	HHLE1_206509_eIF4AIII_rep1_206509_32_152	100.00	152	0	0	1	152	0	+392	Downstream
hsa_circ_0055412	HHLE1_206515_eIF4AIII_rep1_206515_3_43	100.00	43	0	0	1	43	-205	-163	Upstream
hsa_circ_0055412	HHLE2_673405_eIF4AIII_rep2_673405_41_137	100.00	137	0	0	1	137	0	+377	Downstream
